# Decoding THz‐Driven Dynamic Fingerprints of Ferroelectric Nanotwin Networks

**DOI:** 10.1002/adma.73118

**Published:** 2026-05-02

**Authors:** Xiaojiang Li, Aiden Ross, Vladimir A. Stoica, Sujit Das, Sankalpa Hazra, Huaiyu (Hugo) Wang, Hari Padma, Matthias C. Hoffmann, Patrick Kramer, Sanghoon Song, Silke Nelson, Takahiro Sato, Diling Zhu, Ramamoorthy Ramesh, Lane W. Martin, Yue Cao, John W. Freeland, Aaron M. Lindenberg, Haidan Wen, Long‐Qing Chen, Venkatraman Gopalan

**Affiliations:** ^1^ Department of Materials Science and Engineering and Materials Research Institute The Pennsylvania State University University Park Pennsylvania USA; ^2^ Advanced Photon Source Argonne National Laboratory Lemont Illinois USA; ^3^ Materials Research Centre Indian Institute of Science Bangalore India; ^4^ Linac Coherent Light Source SLAC National Accelerator Laboratory Menlo Park California USA; ^5^ Department of Materials Science and Engineering & Department of Physics University of California Berkeley California USA; ^6^ Materials Sciences Division Lawrence Berkeley National Laboratory Berkeley California USA; ^7^ Rice Advanced Materials Institute and Department of Materials Science and NanoEngineering Rice University Houston Texas USA; ^8^ Materials Science Division Argonne National Laboratory Lemont Illinois USA; ^9^ Stanford Institute for Materials and Energy Sciences SLAC National Accelerator Laboratory Menlo Park California USA; ^10^ Department of Materials Science and Engineering Stanford University Stanford California USA

**Keywords:** ferroelectrics, nanodomain network, optical second harmonic generation, THz dynamics, ultrafast X‐ray diffraction

## Abstract

Ultrafast polarization dynamics in ferroelectrics are of considerable interest for high‐speed tunable dielectrics and electro‐optics. Extended domain wall networks formed in ferroelectric twin nanodomains can support collective dynamics in the terahertz regime but require techniques that track polarization and strain evolution driven by ultrafast stimulus. Here, we use multi‐modal probing of THz‐pulse‐driven excitations in PbTiO_3_/SrTiO_3_ superlattices by combining X‐ray free electron laser measurements that directly tracks lattice changes, with optical second harmonic generation that tracks the electronic potential coupled with the lattice potential. Dynamical phase‐field modeling enables fingerprinting of these collective modes as superpositions of domain “breathing” through wall oscillations and polarization “rotations” with still walls. Ultrafast domain wall motion at 0.1–0.5 THz is observed at practical fields of 100 kV/cm with wall velocities of >4000 m/s, approaching typical speed of sound in PbTiO_3_. A unique “charging” mode is discovered that can electrically charge and discharge domain walls on ∼4 ps time scale thus dynamically tuning wall conductivity. Integrated experimental and theoretical fingerprinting of the dynamical landscape presented here enables ultrafast control of ferroics for high‐speed microelectronics and optical applications.

## 1 Introduction

Understanding and controlling the dynamics of ferroelectric domain walls can provide ultrafast functionalities in nanoelectronics applications while harnessing low‐dimensional ferroelectric nanostructures [1, 2, 3, 4]. The properties of various material systems are defined not only by their composition, but also by the nanoscale arrangement and collective dynamics of the ferroelectric domains and domain walls. With increasing demands for high‐speed, high‐performance devices, the behavior at ultrafast timescales becomes ever more important. For example, the dynamic motion of ferroelectric domains allows for high‐frequency tunability and low loss in the ∼10 GHz regime for 5G applications [5, 6, 7], strong electro‐optic response above 100 GHz in silicon photonics [8, 9], and provides control of negative capacitance for low power consumption in transistor circuits [10, 11, 12]. Furthermore, ultrafast ferroelectric switching behavior can enable non‐destructive read‐out and multistate switching for ferroelectric memory applications [13, 14, 15], with potential extension to high‐frequency regimes. To establish deterministic control of nanoscale‐ferroelectric structures, we must develop a fundamental understanding of their ultrafast responses under external stimulus [16]. First, we require methods of control and characterization of dynamics in ferroelectric nanostructures at the fastest possible timescales. Second, modeling and simulation methods are required that can accurately describe the ferroelectric heterogeneous dynamics initiated under external stimulus.

Toward these goals, the (PbTiO_3_)*_n_*/(SrTiO_3_)*_n_* (PTO‐STO) superlattices (where *n* = 4–20 unit cells typically) provide a rich platform for studying collective dynamical responses that are generated from an interplay between polarization and strain heterogeneities at <10 nm length scales. With a variation of *n*, this system supports a variety of phases including vortex, skyrmion, dipolar wave, and supercystal phases which often compete and coexist with conventional ferroelectric twin phases, or so‐called *a_1_/a_2_* ferroelectric nanotwin domain structures [17, 18, 19, 20, 21]. By exciting these extended supertextures with a THz wave, the collective polarization dynamics in nanoscale polar structures such as skyrmions and vortices have been predicted [22, 23] and observed in experiments [24, 25]. The *a_1_/a_2_* ferroelectric nanotwin domains are ubiquitous in ferroelectrics, however, their ultrafast collective dynamical properties remain unexplored. We experimentally report and decode, for the first time, the domain wall collective modes in such *a_1_/a_2_* domain structures. The work also, for the first time, combines THz‐pump and optical second harmonic generation (SHG) probe with X‐ray free‐electron laser (XFEL) diffraction to decode a complementary set of collective modes. We discover a unique domain wall charging mode and identify large domain wall velocities approaching acoustic speeds.

**FIGURE 1:**
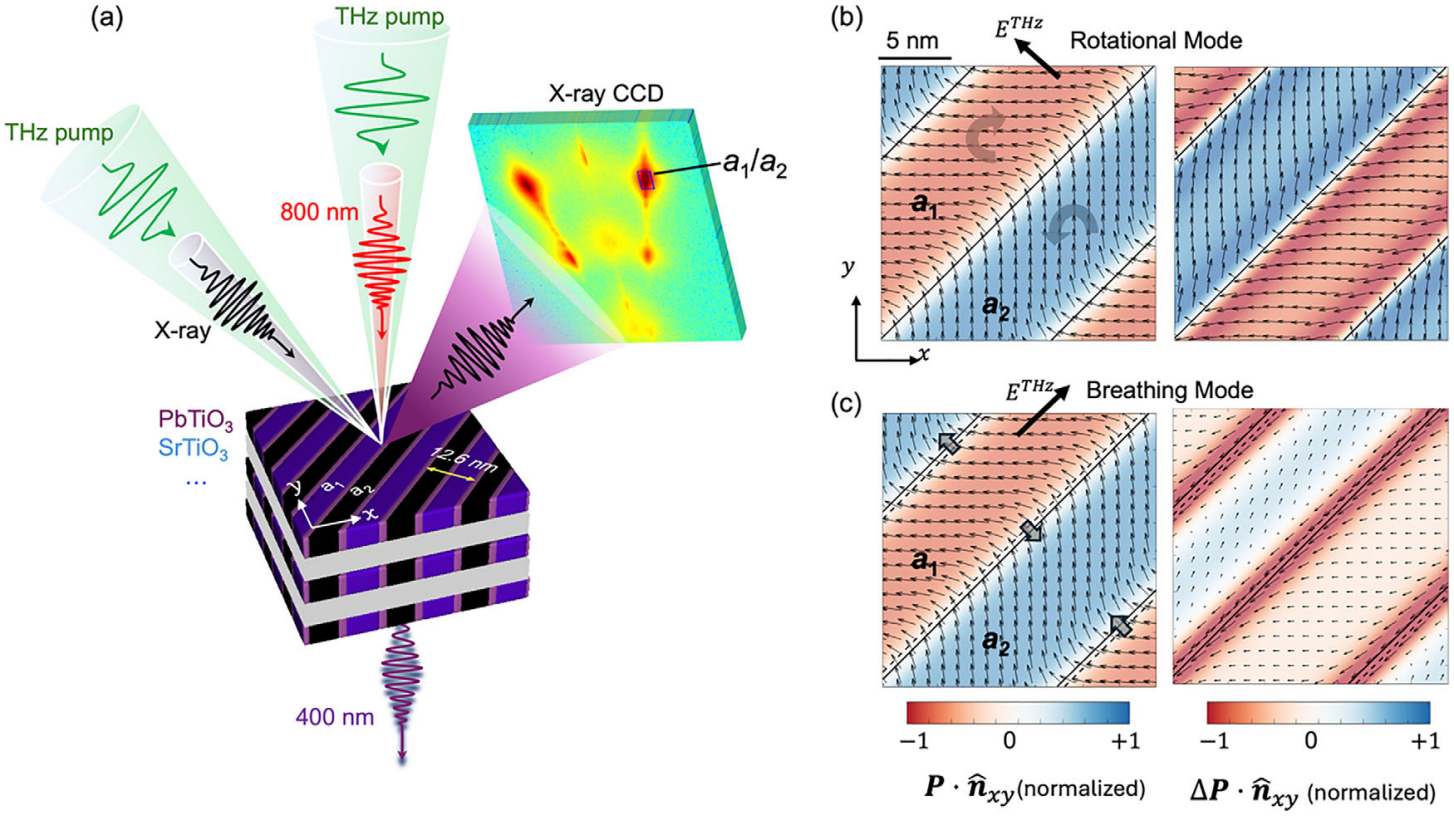
Experimental setup and collective response types to terahertz field excitation (a) Schematic illustration of the time‐resolved THz‐pump, X‐ray probe and THz‐pump, optical SHG‐probe, used to characterize the ultrafast dynamics of ferroelectric *a_1_/a_2_* twin domain microstructure in the PbTiO_3_/SrTiO_3_ superlattices. Dynamic phase‐field modeling (DPFM) of the polarization dynamics in (b) a rotation mode response promoted by THz field perpendicular to the 90° domain wall, and (c) a breathing mode response promoted by THz field parallel to the 90° domain wall. The left panel in each of the modes in b,c) represents the field of polarization vectors, *P_S_* and the right panel represents the field of change in the polarization vector, Δ*P_S_*, upon application of the THz field.

The PTO‐STO superlattices exhibits intricate interactions among domain walls, strain, polarization, charge ordering, and phonons that can be accessed in experiments [21]. Here, we combine ultrafast THz‐pumping with probes of optical SHG and XFEL probes to study the single‐cycle THz pulse excitation of ferroelectric *a_1_/a_2_* domain networks in PTO‐STO superlattices (Figure 1a). To date, linear optical spectroscopy probes of the lattice dynamics of ferroelectric superlattices, such as transient reflectivity, could only capture the acoustic phonons of superlattice heterostructures [26] but remained weakly sensitive to polarization dynamics [27, 28, 29]. In contrast, SHG probes are highly sensitive to broken inversion symmetry, and hence the ultrafast polarization dynamics [22, 23, 24, 25, 29, 30, 31, 32, 33, 34, 35, 36]. XFEL probes, on the other hand, are highly sensitive to core electron motions, allowing it to capture strain and polarization dynamics and can distinguish contributions from different domains in reciprocal space [36]. Since the strain and polarization are coupled through electrostriction, we will show how both these probes become sensitive to polarization and strain dynamics and complement each other in a unified picture of the collective modes. The collective modes distinguished in experiments are fingerprinted by dynamical phase‐field modeling (DPFM) based on the Klein‐Gordon equation for polarization dynamics [37, 38].

## 2 Multimodal Probing of (PbTiO_3_)*_n_*/(SrTiO_3_)*_n_* Heterostructures

The superlattice sample selected for this study consists of a 16 × 16 unit cells (u.c.) PTO‐STO bilayer, repeated eight times, and grown on an orthorhombic (subscript O) (110)_O_ TbScO_3_ (TSO) and (110)_O_ DyScO_3_ (DSO) substrates, with in‐plane axes defined as $x||{\left[\bar{1}10\right]}_{O}$ (lattice parameters 3.960 Å (TSO) and 3.952 Å (DSO)) and *y*||[001]_*O =*_ (lattice parameters 3.959 Å (TSO) and 3.94 Å (DSO)) [39] and the out‐of‐plane substrate direction *z*||[110]_O_ (Figure 1b,c and methods). The PTO‐STO films on TSO exhibit pure ferroelectric *a_1_/a_2_* twin single‐phase configuration. This is in contrast to the PTO‐STO films deposited on orthorhombic DyScO_3_ (DSO) substrates where it coexists with the vortex phase. A ferroelectric polarization hysteresis loop, however, is shown in Figure S1 along with X‐ray diffraction signature of the *a_1_/a_2_* phase, showing consistency in this phase between DSO and TSO substrates [20, 24, 36]. In this work, the collective dynamics of the *a_1_/a_2_* phase is also shown to be consistent between the films on these two substrates. The ±*a*_1_ and ±*a*_2_ tetragonal domains have the tetragonal axis oriented along ±*x* and ±*y*, respectively. The *a_1_/a_2_* nanotwin structure is thus characterized by two in‐plane tetragonal domains, with orthogonal spontaneous polarization *P_S_* along the ±*x* and ±*y* directions arranged in a head‐to‐tail fashion and separated by a 90° domain walls, where the two domains interact via ferroelastic interactions coupling strains across the wall. Depending upon the direction of polarization, *P_S_* in each domain, there can be four different variants of the ±*a*_1_/±*a*_2_ nanotwin structures, namely *P_S_* along the substrate ±*x* or the substrate ±*y* directions; these four variants are dubbed as “domain bundles.” A nearly unipolar ∼1 ps wide THz pulse (Figure S2) is incident on the *a_1_/a_2_* nanotwin domain microstructure and can be applied with the THz polarization parallel to, perpendicular to, or at ±45° to the 90° domain walls (Figure S3). Depending on the selected sample geometry, a time domain response and the corresponding fast Fourier transformation (FFT) spectrum are obtained and used to identify characteristic modes in the polarization dynamics.

Depending on the orientation of THz electric field, distinct dynamical modes can be excited. When the THz electric field is perpendicular to the ferroelectric domain wall, each domain is equally aligned with the THz electric field causing the polarization within each domain to symmetrically rotate toward the THz field direction resulting in a predominant “rotation mode” with minimal domain wall motion (Figure 1b). When the THz electric field is parallel to the domain wall (Figure 1c), however, the ferroelectric polarization in one domain is more aligned with the THz field causing that domain to grow at the expense of the other domain, resulting in a “breathing mode” which is dominated by domain‐wall motion. Depending on the polarization of the THz field, the observed modes may not be “pure” breathing or rotation modes but can have a mixed character of the two. Next, we describe experimentally how to probe the DPFM‐predicted collective modes, first with THz‐pump SHG probe, followed by THz‐pump/X‐ray probe.

### 2.1 Ultrafast Time‐Domain Optical Second Harmonic Generation

**FIGURE 2:**
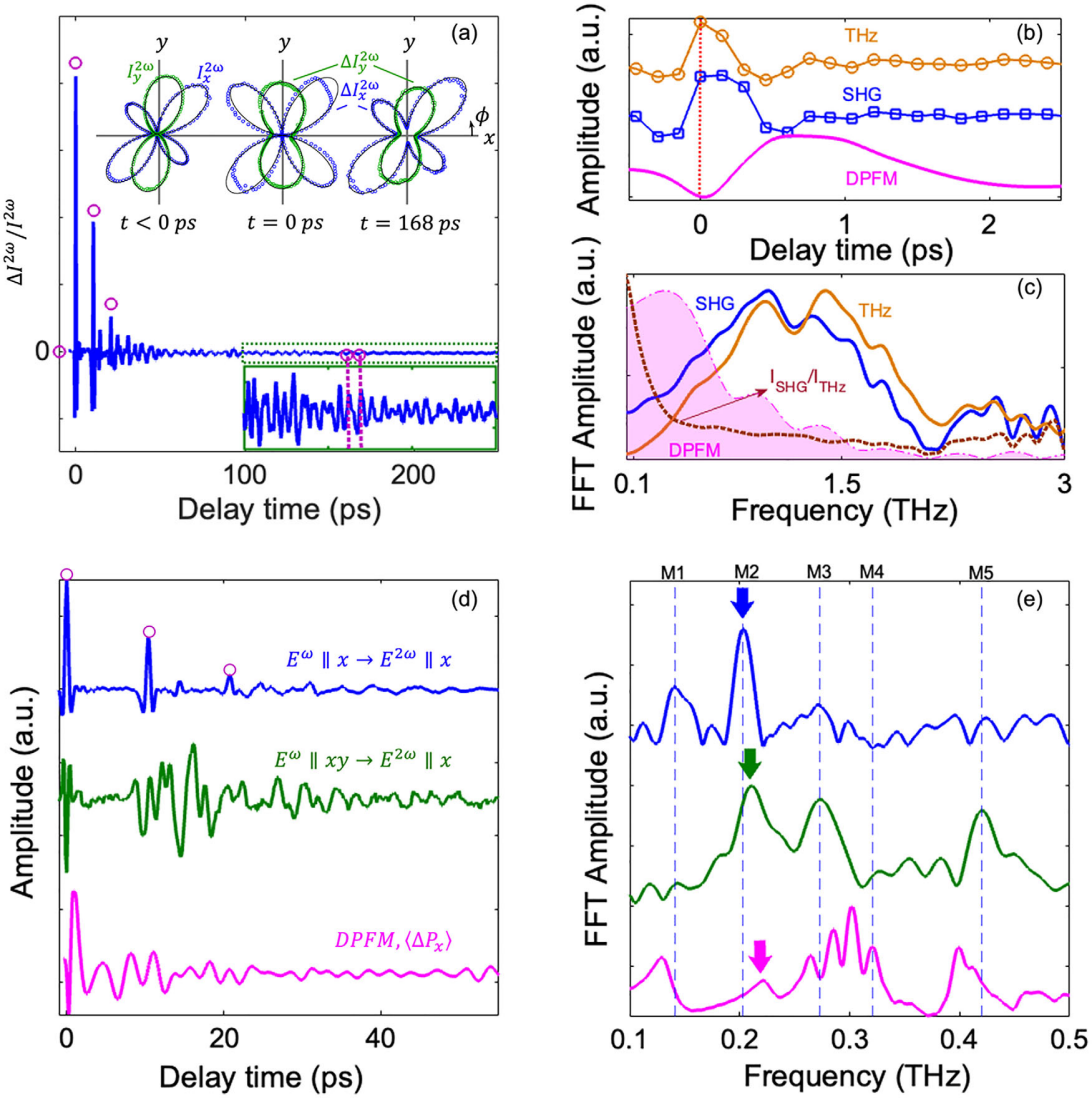
Second harmonic generation from the PTO‐STO superlattice on TSO substrate and dynamic phase‐field model (DPFM) simulations probing the response to THz field excitation polarized in the ***x***‐direction. (a) Time‐resolved THz‐pump SHG probe measurements (blue curve) of ***ΔI*** ^2 ***ω***^/***I*** ^2 ***ω***^ when the THz field ***E*** ^***THz***^∥ ***x***, and the fundamental and SHG fields, ***E*** ^***ω***^, ***E*** ^2 ***ω***^∥ ***x***. SHG polar plots (top insets) were measured at the corresponding locations of the purple circles by varying the angle ϕ of the fundamental ***E*** ^***ω***^ while detecting ***E*** ^2 ***ω***^∥ ***y*** (light green) or ***E*** ^2 ***ω***^∥ ***x*** (blue). The first polar plot is ***I*** ^2 ***ω***^, while the other two are ***ΔI*** ^2 ***ω***^ induced by the THz field. A zoomed‐in view inside the green dotted box is shown in the lower inset. Modeling of polar plots is described in Note S3. (b) The first few ps depict the time trace of THz, SHG, and the spatially averaged polarization response, 〈 ***ΔP***_***x***_〉 from DPFM (curves are shifted vertically for clarity). (c) The corresponding frequency‐domain spectra of (b) along with the ratio of FFT amplitudes of the SHG to the THz signals are shown. (d) The first 50 ps of the SHG time trace for two different polarization geometries (blue and green), along with the DPFM calculation (pink) of the spatially averaged 〈 ***ΔP***_***x***_〉. The purple circles indicate THz echoes. (e) The FFT of the time traces in (d), with five collective modes labeled M_1_–M_5_ that DPFM predicts. All but M_4_ are seen in the SHG experiments. The dominating a_1_/a_2_ nanotwin collective mode at 0.21THz is marked by the color‐coded arrows.

The time and frequency domain responses from THz‐pump SHG‐probe of the PTO‐STO superlattices on TSO are shown (Figure 2). Optical SHG is a nonlinear optical process by which light fields at frequency ω (wavelength of 800 nm) combine in a nonlinear potential well to create a nonlinear polarization at frequency 2ω (wavelength of 400 nm), given by $P_{i}^{2\omega }=d_{ijk}\;E_{j}^{\omega }E_{k}^{\omega }$, where *i, j* and *k* are dummy variables, each indicating any of the crystal physical directions 1, 2, and 3. Since *d_ijk_* is non‐zero in non‐centrosymmetric materials, SHG is sensitive to broken inversion symmetry arising from polarization in materials. The SHG measurement captures the reversible dynamics of the *a_1_/a_2_* nanotwin microstructure (Figure 2a). A strong sub‐ps SHG response is observed that can be compared with the THz pulse waveform and the THz frequency spectrum (Figure 2b,c) followed by several successive echoes of the THz pulses (at time delays of ∼10 and 20 ps; shown by purple circles in Figure 2a,d, which propagate between the two surfaces of the sample and modulate the SHG response at early time delays). These echoes are consistent with the calculated THz pulse roundtrip propagation time through the TSO substrate, where the time delay of reflected THz pulses is given by the refractive index of the TbScO_3_ substrate in the THz range [40] (See Note S1).

The time traces of the THz pulse, the SHG response and the DPFM polarization dynamics are shown (Figure 2b). This sub‐ps response is attributed to THz electric‐field induced second‐harmonic generation (EFISH) effect perturbing the nonlinear polarization, *P^2ω^* and since *I* ^2ω^∝(*P* ^2ω^)^2^, the intensity of the SHG is $I^{2\omega }\propto {\left(\chi^{\left(2\right)}+\chi^{\left(3\right)}E^{THz}+\chi^{\left(4\right)}{\left(E^{THz}\right)}^{2}\cdots \right)}^{2}{\left(I^{\omega }\right)}^{2}$, where χ^(2)^, χ^(3)^ and χ^(4)^ are second, third order and fourth order nonlinear optical susceptibilities of the material and *I* ^ω^ is the intensity of the fundamental laser beam generating SHG, whereas *E^THz^* is the absolute value of THz electric field [41]. It follows that the leading term generating the EFISH intensity, $\Delta I^{2\omega }\propto 2\chi^{\left(2\right)}\chi^{\left(3\right)}{\left(I^{\omega }\right)}^{2}E^{THz}+\cdots$, can be described by linear scaling with *E^THz^* [42]. Here, we choose to carry the measurements at maximum experimental field (∼100 kV/cm) to improve the signal‐to‐noise ratio in polarimetric SHG measurements. The EFISH time‐domain signal is converted to frequency domain using FFT and rescaled for comparison to the maximum amplitude of the FFT corresponding the electro‐optic sampling of the *E^THz^* (Figure 2c and Methods), indicating that above ∼0.5 THz, the SHG response tracks the *E^THz^* spectrum, supporting that it is dominated by EFISH scaling with *E^THz^*. At < 0.5 THz, however, the ratio between the FFT's of the SHG response and *E^THz^* increases, overlapping with the spectral region (pink) where the DPFM predicts collective modes (See Note S2 and Figures S3 and S4).

The SHG polarimetry (Figure 2a inset), captures different representative time instants, and was collected by varying the incident polarization (rotation angle *ϕ* from the lab *x* axis in the *x‐y* plane) of the fundamental field with optical frequency *ω* for different fixed polarization directions (*x*, *y, xy,* $\bar{x}y$ etc.) of 2*ω*, at the second‐harmonic frequency. Modeling of this polarimetry (Note S3 and Figures S5 and S6) at time *t* <0 (before the THz pulse arrival) confirms the 4*mm* tetragonal symmetry with a multidomain domain nanostructure composed of *a_1_/a_2_* nanotwins. At the time instances of each of the three SHG peak responses corresponding to the THz pulse and its echoes (purple circles), however, the differential SHG polarimetry (Δ *I* ^2ω^ = *I* ^2ω^ (*t*) − *I* ^2ω^(*t* < 0)) reveals a single domain like 4*mm* symmetry (Figure S6) with the polar four‐fold axis along the THz polarization direction (*x*‐axis). This result suggests a uniform THz‐induced dynamic poling effect overlaid over the underlying static multidomain *a_1_/a_2_* nanotwin domain structure. Such an EFISH effect could potentially be attributed to the *x*‐polarized THz electric field that impulsively displaces the electronic distribution in the *x*‐direction at a frequency of Ω ≈ 10^12^ Hz, which in turn modulates the optical nonlinearity of the electronic energy well. This change in the potential seen by the electrons in the presence of THz has a single domain 4*mm* symmetry and it generates the corresponding change in the optical SHG reflecting the renormalized electronic potential defined by the THz pulse. The underlying ionic lattice however remains multidomain on this sub‐ps time scale as seen by X‐ray later, indicating that SHG probe of sub‐ps domain switching could be predominantly electronic in nature.

To clarify the dynamics of collective modes here, occurring on tens to hundreds of ps timescales, in the following we focus on the understanding of experimental observation assisted by DPFM simulations (Methods). Since the THz echoes complicate the extraction of the frequency‐domain analysis from the observed time‐domain dynamics due to an interference and cross‐cancellations of multiple forward and backward source waves generating SHG; for collective mode analysis, we focus on time scales after ∼20 ps where clear coherent oscillations are observed (dotted green box, Figure 2a). Two different SHG measurements with different probe SHG polarization configurations are plotted (Figure 2d) along with the temporal evolution of the simulated polarization dynamics, Δ*P_x_* from DPFM. FFT's of these time domain traces are shown (Figure 2e). From the modeling, the SHG time trace $E_{x}^{\omega }\to E_{x}^{2\omega }$ should be sensitive to the breathing modes that try to “pole” the ferroelectric *a_1_/a_2_* phase along the THz polarization direction, *x*, while the SHG time trace labeled $E_{xy}^{\omega }\to E_{x}^{2\omega }$ should be sensitive to both the breathing modes and the rotation modes described before (Figure 1). Four collective modes are observed in the SHG FFT traces: 0.13THz (M_1_ mode), 0.21THz (M_2_ mode), 0.27 THz (M_3_ mode) and 0.42THz (M_5_ mode), which are associated with the modes also seen in the polarization dynamics predicted by the DPFM as it will be discussed later. We will show that M_1_ is predominantly a breathing mode, while M_3_ and M_5_ are predominantly rotation modes, and M_2_ consists of mixed breathing and rotation dynamics. The M_4_ mode is predicted by the DPFM but is not discerned in the measured SHG signal. The dominant modes M_1_‐M_3_ were consistently observed across multiple measurements on the same sample at different spots as shown in Figure S7.

### 2.2 Ultrafast Time‐Domain X‐Ray Diffraction

**FIGURE 3:**
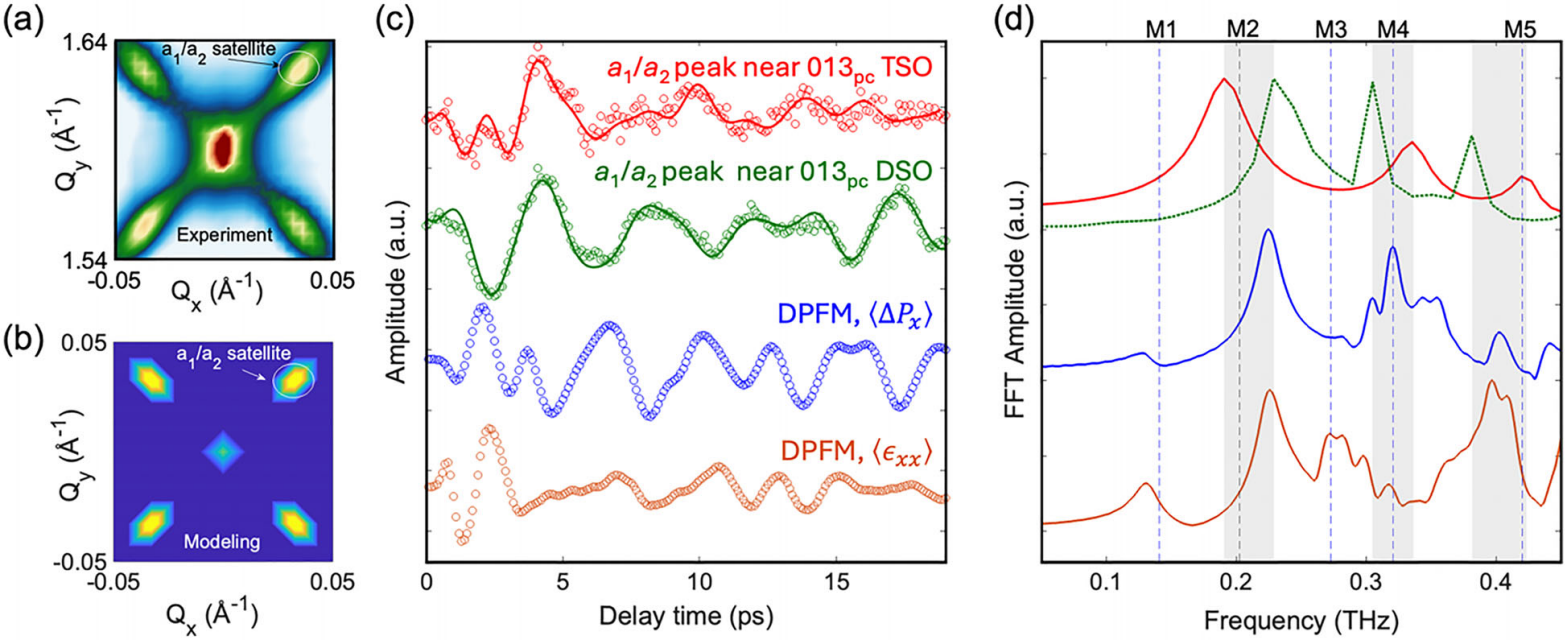
Experimental X‐ray free electron laser (XFEL) observation of collective modes of a_1_/a_2_ microstructures compared with dynamical phase‐field model (DPFM) simulations under a THz field in the ***x***‐direction. (a) a_1_/a_2_ nanotwin diffraction pattern in PTO‐STO superlattices from RSM and (b) FFT of DPFM, where satellite peaks corresponding to the periodic a_1_/a_2_ structure are marked by arrows. (c) Time‐resolved XFEL measurement (red open circle for film on TbScO_3_, green open circle for film on DyScO_3_) on satellite peaks marked in a, b, and the fitting results (red and green solid lines). Time‐resolved dynamics of spatially averaged polarization (blue open circle) and strain (yellow open circle) components of the first‐order satellite from DPFM simulation indicated in panel (b). (d) Frequency‐domain comparison of the modes among the XFEL measurement, fitting, and the DPFM simulations in (c). The shaded areas provide a confidence interval for each mode frequency that is compared with the DPFM modes observed in FFT plots of strain and polarization dynamics.

On the other hand, THz‐pump XFEL‐probe is sensitive to both polarization and strain dynamics, which in comparison with the DPFM simulations (Figure 3) reveals this M_4_ mode. Experimental X‐ray reciprocal space mapping (RSM) studies of the PTO‐STO superlattices in the equilibrium state at room temperature (Methods) capture the satellite peaks characteristics for the *a_1_/a_2_* nanotwin structure (Figure S8) and compares favorably with a FFT of the simulated structure in real space using DPFM (Figure 3a,b). The satellite peaks, experimentally located around the zeroth order superlattices peak in the 103 pc ‐Bragg reflection of the substrate, are formed from two kinds of mutually 90^0^ *a_1_/a_2_* nanotwin domain walls and exhibit a four‐fold rotational symmetry. These domain walls have an experimentally observed periodicity of ∼12.2 nm for the *a_1_/a_2_* nanotwin structure on TSO substrate and close to the one observed on DSO substrate (∼12.5 nm) [24]. The THz‐pump X‐ray probe experiment was conducted using the pseudocubic 103 _pc_ (subscript pc indicating peudocubic notation) Bragg reflection with the THz electric field polarized along the [001]_O_ direction of the TSO substrate (*y*‐axis); this coincides with the ferroelectric polarization direction of the *a*_2_ domain and perpendicular to polarization of *a*_1_. From the time‐dependent response of the XFEL measurements (Figure 3c) of diffuse satellite peaks characteristic for *a_1_/a_2_* nanotwins (Figure 3a), we can see that the collective modes dynamics builds up slowly and continues for tens of picoseconds. The extraction of collective modes from different measurements and samples are obtained from time‐domain fitting of the experimental traces (Figure S9), followed by their FFT to generate the frequency‐domain spectra (Figure 3c). In this model, we decompose the model fitting into four damped harmonic oscillator modes with distinct frequencies.

In addition to *a_1_/a_2_* nanotwin structures on TSO, we have also plotted for comparison the prior measurement [24] of *a_1_/a_2_* nanotwin structures on DSO substrate to inspect the collective mode differences on these two substrates. In this comparison, the main sources of uncertainty expected to modify the frequency of the collective modes are the substrate strain mismatch between PTO/STO superlattices and the TSO and DSO substrates that include ±5% thickness variations of PTO and STO layers across the samples surface due to variations related to laser deposition geometry. We thus include such sample‐related uncertainties as a proxy for establishing confidence intervals in the identification of collective modes (grey colored boxes, Figure 3d), which is derived from mode frequency differences on TSO and DSO substrates. Furthermore, in the case of PTO‐STO superlattices on TSO substrate used in X‐ray and SHG measurements, the M_2_ and M_5_ modes frequency observed with both probes gives excellent agreement, attesting the accuracy of mode identifications with multi‐modal experimental technique used. In DPFM modeling, on the other hand, the main source of uncertainty is the knowledge of PTO and STO polarization mass densities that were adjusted previously to match the calculated and experimental frequencies of the vortexon mode [24]. For *a_1_/a_2_* nanotwins, we find that the best agreement for the simulated and observed collective mode frequencies (Figure 3c,d) corresponds to polarization mass densities that are ~30% larger than comparing with the vortex phase (Note S4). The resulting frequencies of the collective modes match reasonably well with those of the M_2_, M_4_, and M_5_ modes from DPFM that are extracted from the simulated polarization and strain dynamics. By comparing DPFM with experimental results, it is observed that the M_4_ (breathing mode) is dominant in the FFT of polarization dynamics, the M_5_ (rotation mode) dominates the FFT of strain dynamics, and the M_2_ (breathing plus rotation) has comparable relative intensities in FFT's for both polarization and strain dynamics. In our measurements, the key collective modes are consistently observed across different locations on the sample (Figure S7). However, the peak intensities do exhibit spatial variation. We attribute this primarily to differences in the local domain distribution within the laser probe area, which has a diameter of ∼75 µm, and consequently sensitive to variations in the net polarization sampled within each measurement region.

## 3 Phase‐Field Simulations and Mode Analysis

**FIGURE 4:**
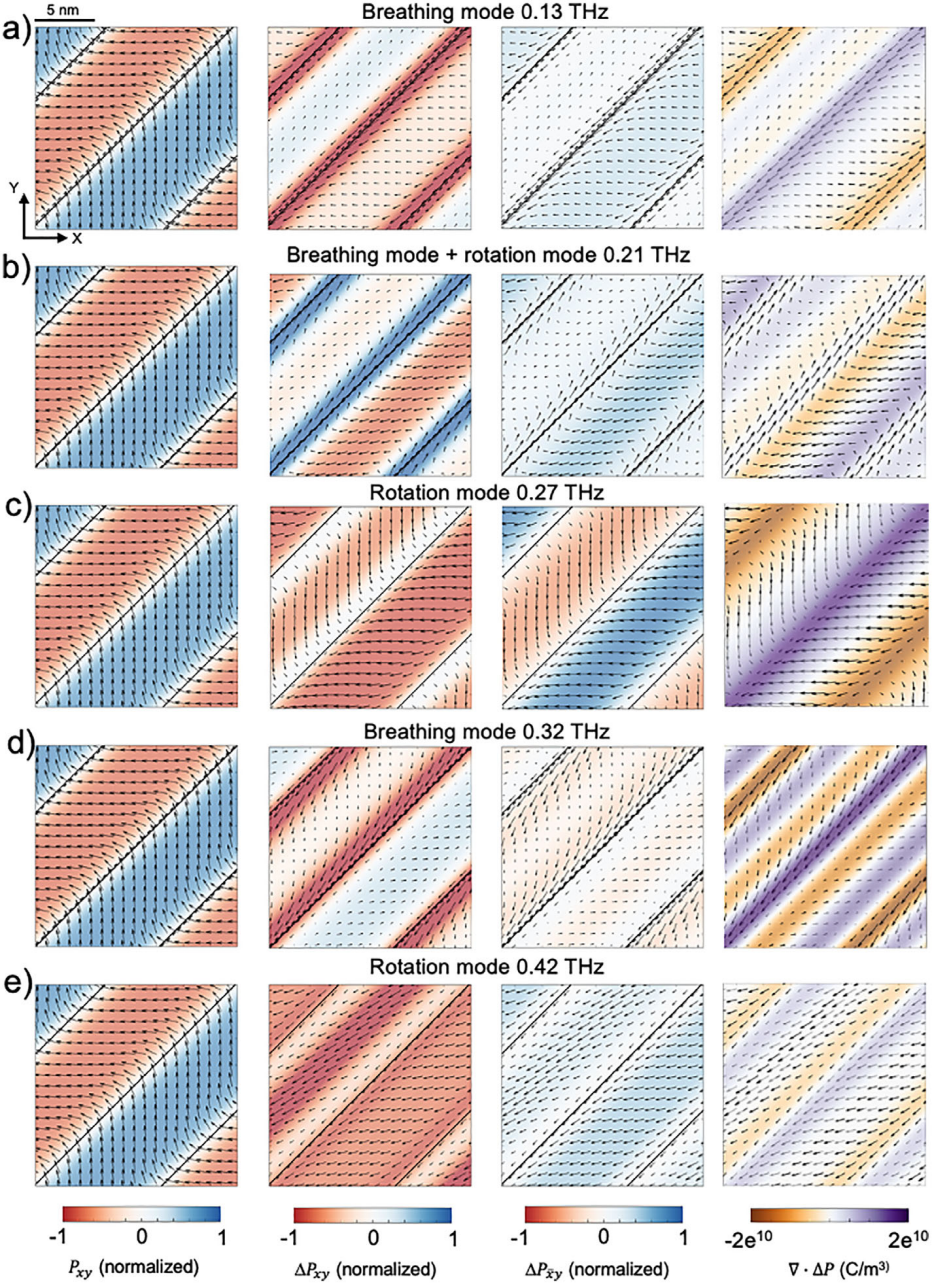
Collective modes from dynamical phase‐field modeling. Three representative excited collective modes resonantly, (a–e), labeled M_1_–M_5_ respectively, predicted by dynamical phase‐field modeling arising from applied THz field in the *x* direction. For each individual mode, the map of the initial polarization, ***P***, along the *xy* direction before the THz field is applied is shown in column 1, while the change in polarization, Δ ***P*** along the *xy* (column 2) and the $\bar{x}y$ (column 3) directions are shown a quarter time period after the THz field pulse is applied, and the dynamical bound charge density ∇ · ΔP (C/m^3^) (column 4). The plots from columns 1 and 2 are colored by the normalized polarization along the [110]_pc_ direction which is parallel to the *a_1_/a_2_* domain wall, while the plots from column 3 are colored by the polarization along the [$1\bar{1}0$]_pc_ direction which is perpendicular to the domain wall. In each plot, the polarization field is normalized by the largest value of polarization or polarization change in the respective maps. The continous lines mark the equilibrium position of domain walls, while the dotted lines mark the domain wall positions under breathing motion induced by THz field.

To better understand the nature of the observed modes, we performed dynamical phase‐field simulations where a single‐frequency AC electric field along the *x* direction, corresponding to the characteristic frequency of each collective mode observed with unipolar THz waveform excitation (Figure S3). This resonant excitation of each collective mode individually isolates the response at the characteristic frequency of interest. The resulting spatially resolved change in polarization (Figure 4) is obtained by comparing the polarization at the oscillation peak with its value a quarter period later using the response at the drive frequency. The resulting spatial distribution of the M_1_–M_5_ modes response reveals regions where the oscillation is strongest in local regions of the nanostructure. We can distinguish the breathing modes driven by domain‐wall motion having an oscillation motion that is concentrated near the domain walls from rotation modes that involve polarization rotation throughout the domains with little to no domain wall motion.

Furthermore, the DPFM simulations reveal distinct mechanisms behind the observed modes with the THz field along *x*‐axis. The M_1_ mode at 0.13 THz and the M_4_ mode at 0.32 THz are primarily breathing modes driven by domain wall motion, where the most marked difference is the phase of domain walls breathing as well as the weaker changes in the *a*_2_ subregions exhibiting opposite polarization changes between these two modes. In contrast, the M_2_ mode at 0.21 THz is a hybrid mode, combining both domain wall motion and polarization rotation.

In the context of ferroelectric switching, large domain wall densities are often associated with increased pinning and disorder which impedes ferroelectric switching. However, the dynamics investigated here do not involve large‐scale domain wall motion but instead arise from collective oscillations of the nanoscale domain structure, about its equilibrium structure. The domain‐wall breathing modes (M_1_ and M_3_) likely have an acoustic‐like dispersion relation, where the eigenfrequency increases with the wavevector. In nanoscale *a_1_/a_2_* domain structures, the spatial periodicity of the domain determines the wave vector of the collective oscillations. As the domain spacing decreases, the wave vector increases, leading to higher resonant frequencies and larger domain wall velocities although the domain‐wall displacements remain small (<2 nm). This mechanism is supported by analytical calculations which show that the resonant frequency of the domain‐wall breathing mode is inversely proportional to the domain wall periodicity [43], as well as atomistic simulations of *c*+/*c*‐ domains showed that the resonant frequency of the collective oscillations of domain wall breathing modes increases as the domain size decreases [16]. Importantly, this collective nanoscale oscillation is fundamentally different from the large‐scale domain‐wall motion involved in ferroelectric switching, where the ultrafast response reflects a phonon‐like resonances of the *a_1_/a_2_* domains. We note that the acoustic like dispersion may break down when the domain wall spacing becomes too small, where several mechanisms, such as structural disorder, which may introduce local pining sites that broaden/damp the resonance and domain wall‐wall interactions which add additional complexity to the dynamics. We hypothesize that the observed ultrafast dynamics occur within an intermediate regime where the domain wall periodicity is sufficiently small to elevate the resonant frequency but remain large enough to maintain the coherent collective oscillations without significant disorder induced damping. Our results highlight that resonant polarization dynamics arise from a distinct physical mechanism than ferroelectric switching. The observed ultrafast response does not rely upon large‐scale domain‐wall motions but collective oscillations of the ferroelectric domains.

The M_3_ mode at 0.27 THz is unique because its spatial distribution shows transient polarization changes that form head‐to‐head and tail‐to‐tail patterns of the polarization (see middle panel in Figure 4c), leading to a dynamical change to the bound charge quantified by Δ ρ_*b*_ =   − ∇ · (Δ*P*) where Δρ_*b*_ is defined by the change in the bound charge due to the coherent mode. (A weaker charging effect exists in other modes as well). To further interrogate the nature of the M3 mode, future work could investigate its coupling with charge carrier dynamics in the system. For example, future experiments could combine an optical pump that modulates the free carrier density with a THz‐pump and SHG‐probe to create a systematic analysis of how carrier injection modifies the behavior of the M3 oscillations.

The M_5_ mode at 0.42 THz appears to involve nearly uniform polarization rotation throughout all domains in the film. To enrich further the visualization of heterogenous polarization texture responding to the THz field applied along and perpendicular to domain walls (Figure S10), we confirm further that susceptibility to polarization rotations can be enhanced for several modes when the field direction is perpendicular to the domain walls. These results reinforce that transient polarization rotations found in densely packed ferroelectric nanostructures take a dominant role in the THz‐field‐induced dynamics.

To review the observed collective modes from the multimodal approaches, we list M_1_‐ M_5_ modes explored under different conditions (Table 1). One can immediately see that different experimental geometries and probe techniques are sensitive to different collective modes. We first note that SHG detects changes to the nonlinear electronic potential; on sub‐ps time scales, it is dominated by the THz‐induced SHG changes in response to the renormalized electronic potential, and over longer time periods, it can reflect the underlying ionic displacements coupled to the electronic potential. X‐ray diffraction measurements presented here, on the other hand are predominantly sensitive to the lattice periodicities and the consequent changes to ionic polarization and strains. The modes presented in this study are through the FFT of time domain data over tens of picoseconds, and hence both electronic and ionic displacements should be reflected in both probes. However, we find that X‐ray‐based measurements do not observe the M_1_ (0.13THz) and the M_3_ (0.27 THz) modes, while SHG measurements are able to capture them, indicating that lattice changes are weaker in these modes. In contrast, in the XFEL measurements, the first order *a_1_/a_2_* nanotwin satellite peak around the 103 _pc_ and 113 _pc_ Bragg peak is sensitive to the M_4_ mode with breathing character, but the M_4_ mode is not detected in the SHG measurements in both geometries, indicating strong lattice changes, but weaker changes in the nonlinear electronic potential. In the SHG measurements, the probe having incident polarization as $E_{x}^{\omega }$ is sensitive to the M_1_ mode with breathing character and absent with $E_{xy}^{\omega }$ probe polarization, whereas $E_{xy}^{\omega }$ probe is strongly resonant with the M_5_ mode with a rotational character, which is absent with the $E_{x}^{\omega }$ probe polarization. While the absence of modes does not preclude them being observed by a particular experimental technique, it is a matter of the observed signal‐to‐noise ratio as well as the measurement geometry, highlighting the importance of using complementary techniques to fully characterize collective modes in ferroelectrics.

**TABLE 1:** Summary of the observed collective modes for various probe geometries.

Mode	M1: 0.13 THz Breathing	M2: 0.21 THz Breathing + Rotation	M3: 0.27 THz Rotation (Charging)	M4: 0.32 THz Breathing	M5: 0.42 THz Rotation
Probe					
SHG $E_{x}^{\omega }\to E_{x}^{2\omega }$	Observed	Observed	Observed	Not detected	Not detected
SHG $E_{xy}^{\omega }\to E_{x}^{2\omega }$	Not detected	Observed	Observed	Not detected	Observed
*a_1_/a_2_* X‐ray satellite, experiment	Not detected	Observed	Not detected	Observed	Observed

To experimentally uncover the “missing” collective modes between the M3 and M4 modes (0.27‐0.32 THz) in the DPFM curves, several approaches can be employed: using different polarization geometries, extending the temporal scan range, and improving statistical averaging to enhance the signal‐to‐noise ratio. For example, as shown in Figure S11, by adjusting the SHG polarization configuration compared with Figure 2d,e, we can faintly observe a mode situated between M3 and M4. However, this mode is clearly much weaker than the prominent signatures displayed in Figure 2e. Taken together, systematic SHG and X‐ray measurements with refined selection rules and improved statistics could allow the full set of theoretically predicted collective modes to be experimentally observed.

## 4 Conclusion

In summary, the dynamic modalities of ferroelectric nanotwins *a_1_/a_2_* in response to THz excitation identified with X‐ray and SHG probes reveal a diverse set of collective modes of an extended nanotwin domains connected by a domain wall network. Through phase‐field simulations, we confirmed the nature of these modes that distinguishes nanodomain “breathing” and polarization “rotation” degrees of freedom in the dynamics. The dynamical response of the ferroelectric nanotwin structure can vary greatly depending on the dominant mode, indicating possibilities of dynamic electrical charging of the ferroelectric domain walls and extending tunable dielectrics and domain wall electronics toward THz frequencies. DPFM results clearly demonstrate that different order parameters such as polarization and strain have distinct responsiveness to the THz stimulus, inviting a wider exploration of these selection rules to guide future studies. The identification of domain breathing modes at sub‐THz frequencies is at least one order of magnitude faster than prior exploits for high‐frequency dielectric tuning [5, 6] suggesting even faster possible applications than before. Wall velocities in the breathing mode are approaching >4000 m/s (Note S5 and Figure S12) at reasonable field values of 100 kV/cm; as a comparison, typical longitudinal speed of sound in PbTiO_3_ is up to 5600 m/s at room temperature [44]. Local polarization rotations revealed in the *a_1_/a_2_* nanotwins dynamics were not observed previously inside larger ferroelectric domains [37, 45], highlighting the importance of high‐density domain walls in low‐dimensional ferroelectric nanostructures for creating emergent ultrafast polarization dynamics. The collective modes of *a_1_/a_2_* nanotwins could help experimentally realize the predictions of pseudo‐magnetism accompanying the motion of domain walls via $M\;∼\;P\;\times \;\partial_{t}P$ [46]. A stronger THz electric field than in this study could enable nonlinear and ultrafast switching phenomena via excitation of collective modes dynamics in heterogenous ferroelectric structures [33]. Overall, our present observations relying on SHG, XFEL and compared with DPFM, highlight the necessity and significance of having multimodal approaches that can unveil the complexity of collective excitations in ferroelectrics.

## 5 Experimental Section

### 5.1 Sample Fabrication and Nonlinear Optical Measurements

Layers of (PbTiO_3_)*_n_*/(SrTiO_3_)*_n_* (*n*  =  16 unit cells) with ∼100 nm thickness on DyScO_3_ (110) substrate were grown at 620°C and 100 mTorr oxygen pressure using the pulsed laser deposition as described before, generating a phase mix between polar vortex and *a_1_/a_2_* nanotwin domains.^22^ Similarly, the (PbTiO_3_)*_n_*/(SrTiO_3_)*_n_* (*n*  =  16 unit cells) on with ∼ 100 nm thickness were grown on TbScO_3_ (110) substrate, generating a phase pure ferroelectric *a_1_/a_2_* nanotwin phase. For time‐resolved measurements on the samples grown on TbScO_3_ substrate, THz‐pump SHG‐probe: a Ti:Sapphire femtosecond laser followed by TOPAS parametric amplifier from Spectra‐physics was used on the THz‐pump SHG‐probe setup. The single‐cycle terahertz pulse beam was generated by a pulsed laser with 1550 nm wavelength modulated at 500 Hz in a PNPA organic crystal. The vertically polarized THz beam was then expanded to 2 inches in diameter by a pair of parabolic mirrors and focused by a 2‐inch focal length parabolic mirror on the sample with the focal size around 340 µm. The maximum field at the focal point of the THz beam was ∼100 kV/cm, as calibrated by electro‐optic sampling method with a GaP crystal. For the probe beam, we used 800 nm pulsed laser with repetition rate 1 kHz and pulse width 100 fs. The probe beam was colinear with the THz beam after the third parabolic mirror and was focused on the sample by a 20 cm focal length lens with the focal size around 75 µm. The pulse energy of the probe beam was around 250 nJ. The SHG signal from the sample was then detected by a PMT after a 400 nm bandpass filter.

### 5.2 Synchrotron and X‐Ray Free Electron Laser Measurements

Synchrotron X‐ray diffraction (XRD) data were taken at the Advanced Photon Source using 12‐ID‐B beamline of the Advanced Photon Source (APS), Argonne National Laboratory. A Pilatus area detector. XRD data were converted from images taken with a 100k Pilatus area detector into reciprocal space representation using rsMap3D and cut along different directions to create different views of the data. The diffuse scattering satellites (Figure 3a) correspond to in‐plane periodic lattice modulations of the ferroelectric *a_1_/a_2_* nanotwins. Reciprocal lattice units from figures are shown as 2π/*d_hkl_* Å^−1^, where *d_hkl_* is the lattice spacing for the *hkl* reflection. Pseudocubic lattice parameters relative to the orthorhombic DyScO_3_ substrate are shown in the following reference system: [100]_pc_ || [$\bar{1}$10]_o_ and [010]_pc_ || [001]_o_, where the “pc” and “o” subscripts represent the pseudo‐cubic and orthorhombic symmetry, respectively. THz‐pump X‐ray probe: the X‐ray pump probe experiments were conducted in the beamline of the Linac Coherent Light Source (LCLS) at the X‐ray probe (XPP) beamline. The LCLS delivered horizontally polarized X‐ray pulses with a pulse duration of 40 fs at a repetition rate of 120 Hz. Hard X‐ray pulses at 9.6 keV were elected by using a diamond (111) monochromator and focused to ∼200 µm full width at the half maximum beam size with beryllium compound refractive lens. A Ti:Sapphire femtosecond laser system synchronized to the free‐electron laser was used to generate ∼40 fs, 800 nm ultrafast laser pulses with up to 20 mJ per pulse, which were then converted to single‐cycle terahertz pulses through a tilted wavefront method in a LiNbO_3_ crystal. The THz pulses were vertically polarized with a spot size of < 1 mm in diameter and maximum peak electric field of ∼800 kV/cm.

### 5.3 Dynamical Phase‐Field Simulations

We employ the dynamical phase‐field method to simulate polarization and mechanical displacement dynamics in response to the THz field excitation. The evolution of the polarization is governed by the polarization dynamics equation

$$\mu_{ij}\frac{\partial^{2}P_{j}}{\partial t^{2}}+\gamma_{ij}\;\frac{\partial P_{j}}{\partial t}=-\frac{\delta F}{\delta P_{i}}$$

where µ_*ij*_ is the polarization effective mass, γ_*ij*_ is the polarization damping constant and *F* is the total free energy which is expressed as the integral over the following energy densities.

$$F=\int f_{Landau}+f_{Elastic}+f_{Electric}+f_{Gradient}\;dV$$

Following the Landau–Ginzburg–Devonshire Theory of Ferroelectricity [47], the Landau free‐energy density describes the intrinsic stability of the ferroelectric phase with respect to the paraelectric phase and is written as a Taylor expansion of the polarization about the paraelectric phase (i.e. *P_i_* =  0):

$$\begin{array}{ccc} \;f_{Landau} & & =a_{11}\;\left(T\right)\left(P_{1}^{2}+P_{2}^{2}+P_{3}^{2}\right)+a_{1111}\left(P_{1}^{4}+P_{2}^{4}+P_{3}^{4}\right)+\\ & & a_{1122}\left(P_{1}^{2}P_{2}^{2}+P_{2}^{2}P_{3}^{2}+P_{1}^{2}P_{3}^{2}\right)+a_{111111}\left(P_{1}^{6}+P_{2}^{6}+P_{3}^{6\;}\right)+\\ & & a_{111122}\left(P_{1}^{4}\left(P_{2}^{2}+P_{3}^{2}\right)+P_{2}^{4}\left(P_{1}^{2}+P_{3}^{2}\right)+P_{3}^{4}\left(P_{1}^{2}+P_{2}^{2}\right)\right)+\\ & & a_{112233}P_{1}^{2}P_{2}^{2}P_{3}^{2} \end{array}$$

where *a_ij_*, *a_ijkl_*, and *a_ijklmn_* are the dielectric stiffness coefficients measured under constant stress conditions. For PbTiO_3_ a sixth‐order expansion [48] is used and for SrTiO_3_ a fourth‐order expansion is used [49]. The dielectric stiffness coefficients are given in Table S1 (See Supporting Information).

The gradient energy density is

$$\;f_{gradient}=\frac{1}{2}\;G_{ijkl}\frac{\partial P_{i}}{\partial x_{j}}\frac{\partial P_{k}}{\partial x_{l}}$$

where *G_ijkl_* is the gradient energy tensor. The elastic energy density is

$$\;f_{elas}=\frac{1}{2}\;c_{ijkl}\left(\varepsilon_{ij}-\varepsilon_{ij}^{0}\right)\left(\varepsilon_{kl}-\varepsilon_{kl}^{0}\right)$$

where *c_ijkl_* is the elastic stiffness tensor, ε_*ij*_ is the total strain (using the stress‐free paraelectric phase as a reference), and $\varepsilon_{ij}^{0}$ is the eigenstrain which is related to the polarization by

$$\;\varepsilon_{ij}^{0}=Q_{ijkl}\;P_{k}P_{l}$$

where *Q_ijkl_* is the electrostrictive tensor. The elastic stiffness and electrostrictive coefficients are given in Table S1 (see Note S4). For simplicity, we assume the elastic stiffness coefficients are homogenous throughout the superlattice. To calculate the local strain, we use the mechanical displacement (*u_i_*) where the evolution of the mechanical displacement is given by the electrodynamic equation

$$\rho \;\frac{\partial^{2}u_{i}}{\partial t^{2}}=\nabla \cdot \left(\sigma_{ij}+\beta \frac{\partial \sigma_{ij}}{\partial t}\right)$$

where ρ is the material mass density, β is the stiffness damping coefficient and σ_*ij*_ is the stress field.

The electrostatic energy density is

$$\;f_{elec}=-E_{i}P_{i}-\frac{1}{2}\varepsilon_{0}\kappa_{ij}^{b}E_{i}E_{j}$$

where ε_0_ is the vacuum permittivity and $\kappa_{ij}^{b}$ is the background dielectric constant which is chosen as 20 and is isotropic. More details on numerical methods for the dynamical phase field method can be found in the literature [38].

We define the superlattice structure by spatially varying the material properties (i.e., dielectric stiffness coefficient) using a sharp interface description. The interface between the ferroelectric film and the TbScO_3_ (TSO) substrate is treated as coherent allowing for the lattice mismatch strain between the TSO (110) surface and the ferroelectric layer to be calculated using the equivalent cubic lattice constant ($a_{PbTiO3}^{c}=3.9569\;Å$, $a_{SrTiO3}^{c}=3.905\;Å$), and the pseudocubic lattice parameters of TSO $\left(a_{\mathrm{TSO}}^{{\left[100\right]}_{\mathrm{pc}}}=0.5\;\sqrt{a^{2}+b^{2}}=3.960\;Å,\;a_{\mathrm{TSO}}^{{\left[010\right]}_{\mathrm{pc}}}=0.5\;c=3.959\;Å\;\right)$ [39]. Since the lattices of the film and substrate are orthogonal there is no in‐plane misfit shear strain such that:

$$\;\varepsilon_{11}=\frac{a_{\mathrm{TSO}}^{{\left[100\right]}_{\mathrm{pc}}}-a^{c}}{a^{c}}\;,\;\varepsilon_{22}\frac{a_{\mathrm{TSO}}^{{\left[010\right]}_{\mathrm{pc}}}-a^{c}}{a^{c}},\;\;\varepsilon_{12}=\varepsilon_{21}=0$$

A system size of 144Δ*x*_1_ × 144Δ*x*_2_ × 32Δ*x*_3_ is used. The thickness of the PbTiO_3_ and SrTiO_3_ layers are set to 16Δ*x*_3_. Periodic boundary conditions were applied in all directions. In all simulations Δ *x*_1_ =  Δ *x*_2_ =  Δ *x*_3_ =  0.4 *nm*.

To initiate the simulation, a single *a_1_/a_2_* superdomains artificially generated with a periodicity matching the experiment then evolved to reach a steady state condition. To simulate the response to the THz excitation a time‐dependent electric field determined by the experimental EO sampling is added with a maximum amplitude of 100 kV/cm. To study the individual modes, a sinusoidal electric field with the same period of the mode is added to the simulation with a maximum amplitude of 10 kV/cm.

To describe the ultrafast dynamics of the ferroelectric domains, the dynamical phase field method models the coupled evolution of three fields, the polarization field (*P_i_* (*x_i_*,*t*)), the elastic displacement field (*u_i_* (*x_i_*,*t*)),) and the electrical potential field $\left(\phi (x_{i},t)),\;\right)$. The polarization dynamics are governed by a polarization dynamic (Klein–Gordon) equation, which includes both an inertial term $\left(\mu \frac{\partial^{2}P_{i}}{\partial t^{2}}\right)$, where µ is the effective mass of the polarization field, and a viscous damping term $\left(\gamma \frac{\partial P_{i}}{\partial t}\right)$ where γ is the damping coefficient of the polarization field. The elastic displacement simultaneously evolves according to the elastodynamic equation with a similar inertial and damping term. For the electrical potential field, we assume that the electrodynamic field, relaxes much faster than both the polarization and elastic displacement fields, and we therefore approximate the electrical potential using the electrostatic equilibrium (Poisson equation). By solving these coupled equations, the DPFM captures the collective oscillations of the nanoscale ferroelectric polarization and the coupling between the electric and elastic fields.

As in standard phase‐field methods, the polarization and strain are assumed to be continuous order parameters, and interfaces such as domain walls are modeled as diffuse regions. This approximation will coarsely grain over the exact atomic scale details of the of the domain wall structure but represents the energetics and dynamics of the collective domain wall modes.

The polarization dynamic equation is parameterized based upon the behavior of the ferroelectric soft mode. As a result, higher‐frequency optical phonon branches are not included in the dynamics. This limits our description of very high frequency optical modes and their anharmonic couplings to the ferroelectric soft mode, but it does not affect the main conclusions of this work which focuses on the resonant collective motion of the ferroelectric domain structure driven by the soft mode‐dominated polarization dynamics. These approximations may influence the highest accessible frequencies or damping, but they do not alter the mechanisms of the ferroelectric domain wall oscillations.

## Conflicts of Interest

Long‐Qing Chen has a financial interest in MuPRO, LLC, a company which licenses and markets the software package used in this research.

## Supporting information




**Supporting File**: adma73118‐sup‐0001‐SuppMat.docx.

## Data Availability

The data shown in the main figures are available at https://doi.org/10.17605/OSF.IO/23VTU. Any additional data of interest are available from the corresponding authors upon reasonable request.
