# Supplementary material for: Decoding THz‐Driven Dynamic Fingerprints of Ferroelectric Nanotwin Networks
Source: Adv Mater. 2026 May 2;38(32):e73118. doi: 10.1002/adma.73118 (PMC13244814; doi:10.1002/adma.73118)
Supplement: Supplementary file 1 — Supporting File: adma73118‐sup‐0001‐SuppMat.docx. [file ADMA-38-e73118-s001.docx]

**Supporting Information, adma.202517519**

Decoding THz-Driven Dynamic Fingerprints of Ferroelectric Nanotwin Networks

Xiaojiang Li,^#^ Aiden Ross,^#^ Vladimir A. Stoica,*^,#^ Sujit Das, Sankalpa Hazra, Huaiyu (Hugo) Wang, Hari Padma, Matthias C. Hoffmann, Patrick Kramer, Sanghoon Song, Silke Nelson, Takahiro Sato, Diling Zhu, Ramamoorthy Ramesh, Lane W. Martin, Yue Cao, John W. Freeland, Aaron M. Lindenberg, Haidan Wen, Long-Qing Chen, Venkatraman Gopalan*


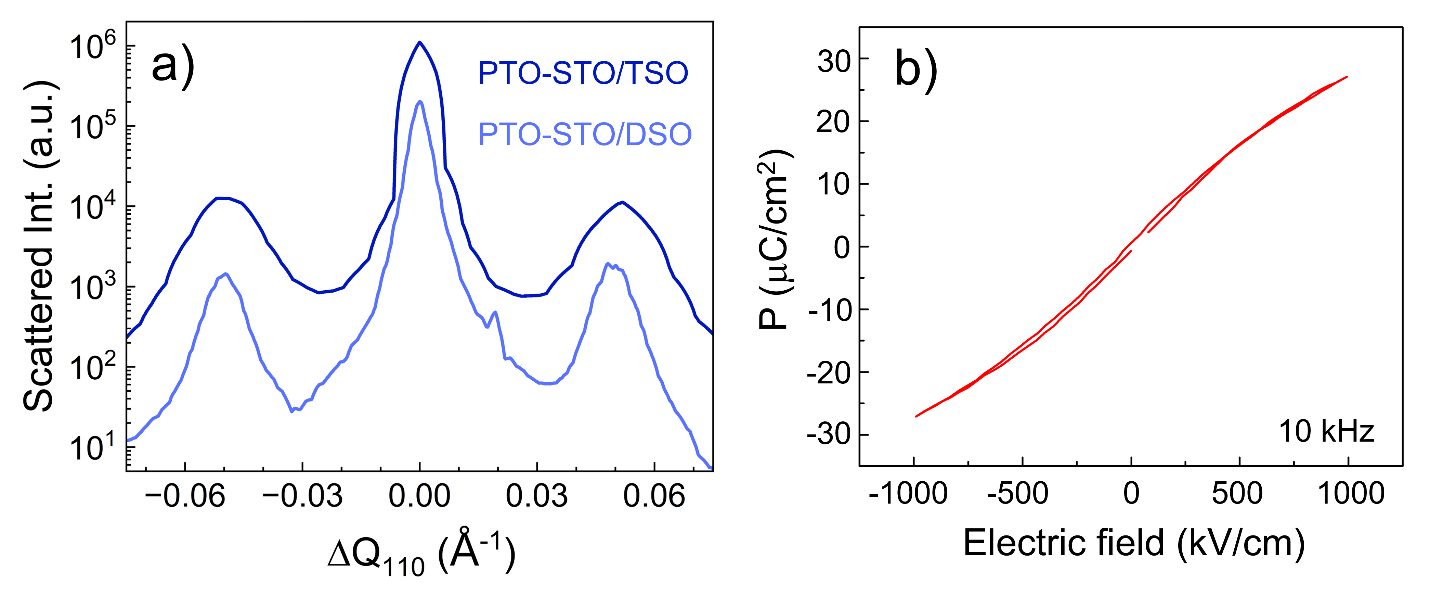


**Figure S1 (a) X-ray diffraction characterization of a_1_/a_2_ nano-twin phase** Line cuts through the diffraction pattern around the 013 pseudocubic Bragg peak for 16 u.c. x 16 u.c. PbTiO_3_-SrTiO_3_ superlattice samples on TbScO_3_ (PTO-STO/TSO) and DyScO_3_ (PTO-STO/DSO) substrates, where the satellite peaks are indicative of periodic a_1_/a_2_ nano-twin phase. The two curves are displaced with an arbitrary offset for better visibility. The line cuts are taken at Q_z_ =4.826 Å^-1^ for PSTO-STO/DSO and Q_z_ =4.831Å^-1^ for PSTO-STO/TSO, both corresponding to *n*=0 superlattice peak of the *a_1_/a_2_* nano-twin phase. The sample on TSO substrate is a pure a *a_1_/a_2_* nano-twin phase. The sample on DSO substrate is a phase mix between the *a_1_/a_2_* nano-twin phase and polar vortices. **(b)** Polarization-electric field hysteresis loop for the (PbTiO_3_)_16_/(SrTiO_3_)_16_ thin film on (110)_O_ DyScO_3_ substrate with field along [100] pseudocubic in-plane direction mixing in contributions from *a_1_/a_2_* nano-twin phase and polar vortices [20]. Due to similarity of *a_1_/a_2_* nano-twin phase for the samples on DSO and TSO substrates as shown in a), indicating the same periodicity, the P-E response of the *a_1_/a_2_* nano-twin phase is expected to be similar for samples on both substrates.

**
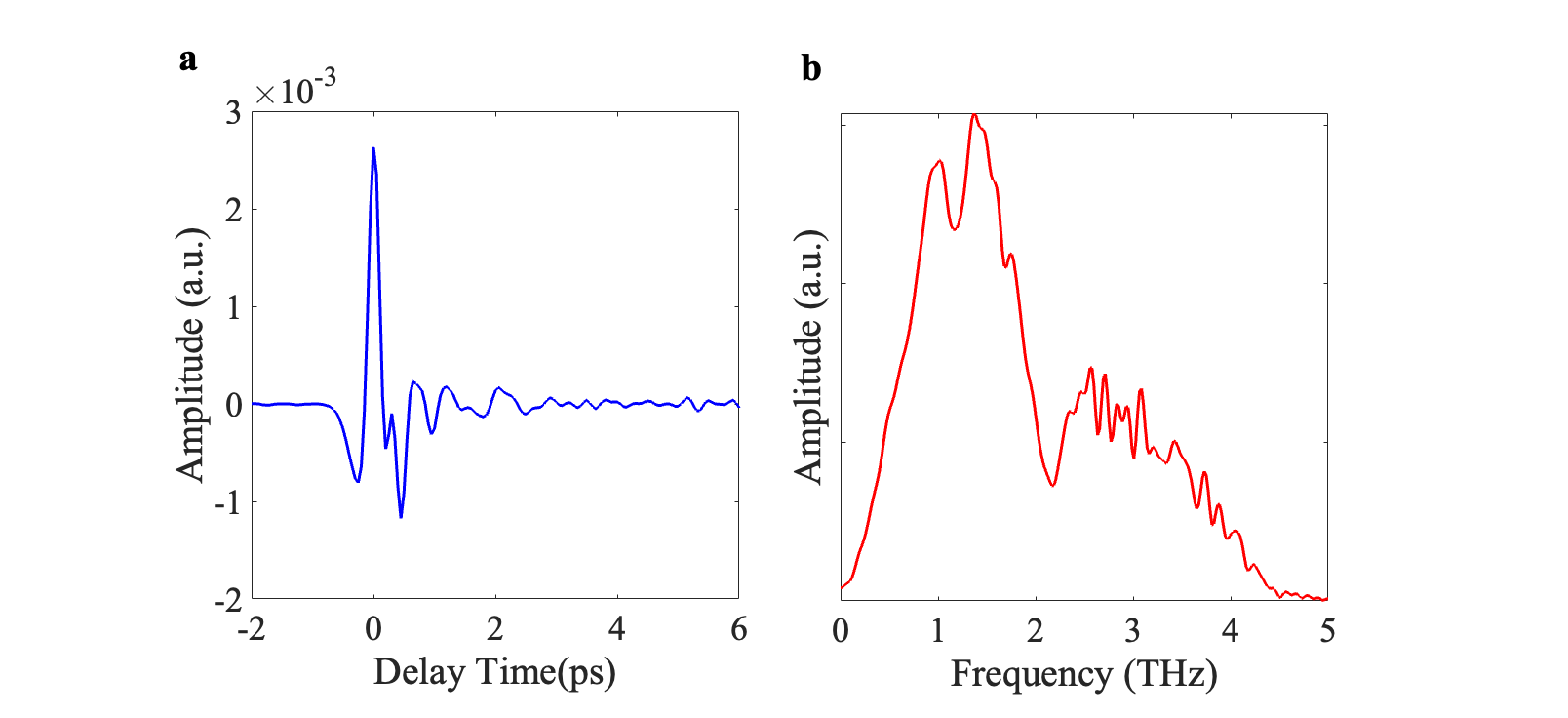
**

**Figure S2 THz pump profile**: The THz pulse is generated by optical rectification of 800 nm pulsed laser in a PNPA organic crystal and measured by electro-optic sampling (EOS) in a GaP single crystal. The time-domain and corresponding frequency-domain spectra are shown in (a) and (b) respectively. The peak field in time-domain is estimated to be 100 kV/cm covering a spectral weight between 0.1 to 4.5 THz in the frequency-domain.

**
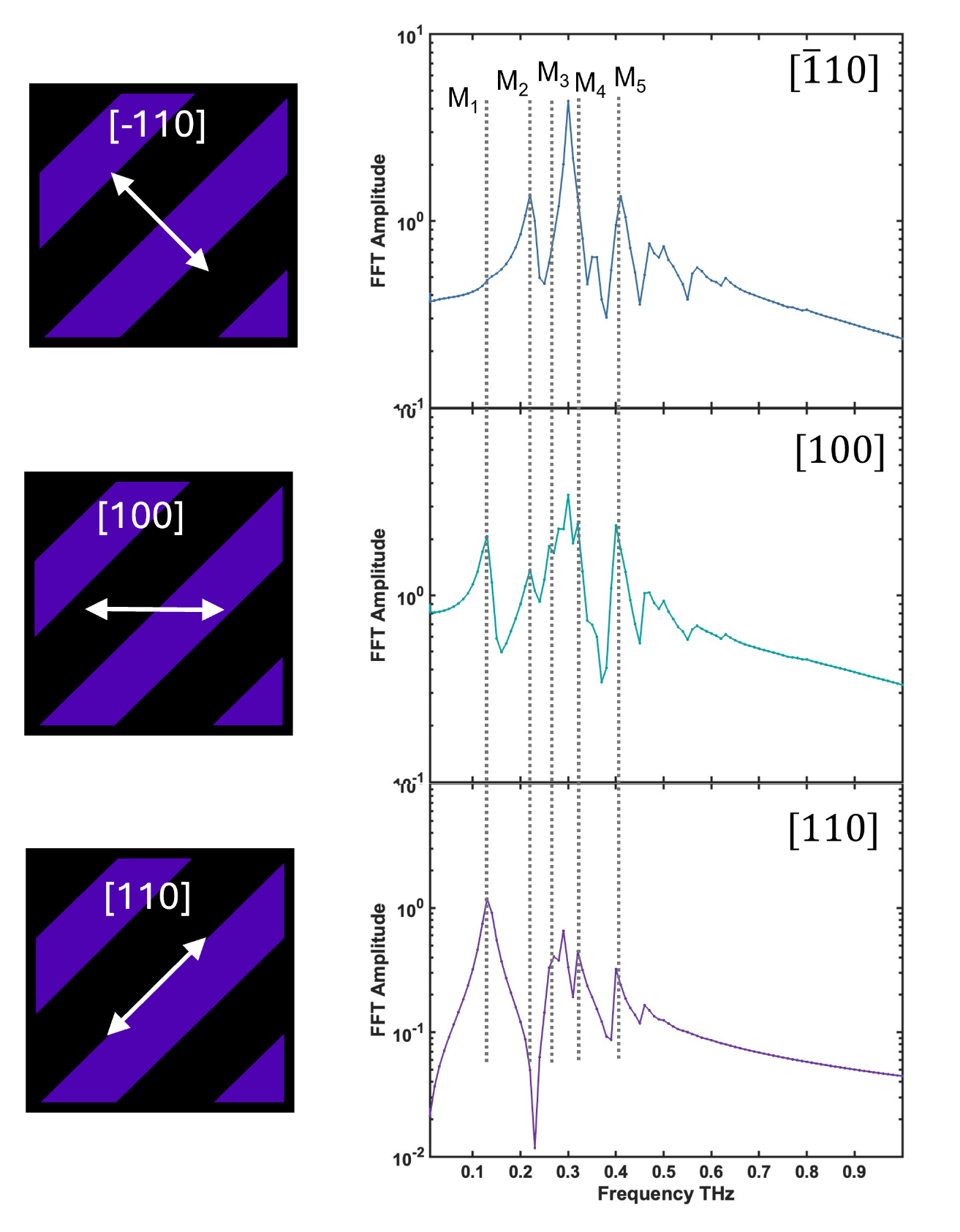
**

**Figure S3 Simulated polarization dynamics in *a_1_/a_2_* nanotwins under THz pulse excitation** The terahertz electric field direction relative to the *a_1_/a_2_* nanotwins structure is shown in the left side column. The FFT of in-plane polarization dynamics resulting from the experimental THz pulse excitation in SHG measurements (~ 100 kV/cm maximum amplitude) is shown on the panels from the right-side column. The M_1_-M modes discussed in the main text are marked with vertical dotted lines

**Note 1: Echoes in THz-pump, SHG-probe Experiments**

The time dependency of THz pulse echoes in SHG measurement from the main text can be estimated as follows: the thickness of the sample (substrate plus the PTO/STO superlattices) is 340 μm, the averaged refractive index at 1 THz is ~ 4, so the time for back reflection is about 10 ps, very close the relative delay time (10.35 ps) between the first and second peak in the time trace of SHG measurement.

**Note 2: Response function of the PbTiO_3_/SrTiO_3_ superlattice film under THz excitation**

**
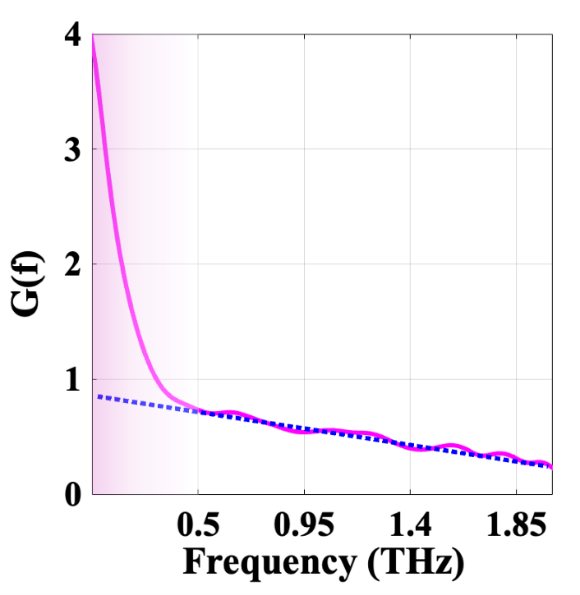
**Convolution theorem: $H\left( f \right)=F\left( f \right)\times G(f)$, where *f* is the frequency, $H\left( f \right)$ is the SHG signal in frequency domain, F(f) is the THz stimulation, then $G(f)$ is the response function of the superlattices.

**Figure S4 Response function of the PTO/STO thin film under THz excitation** The response function $G(f)$ vs. frequency is plotted in pink solid line. The blue dashed line is a linear fitting of the response function above 0.5 THz. Below 0.5 THz, the response function surges up dramatically as compared to the linear fitting, which indicates resonant excitation of collective modes in PbTiO_3_/SrTiO_3_ thin film driven coherently by the THz field.

**Note 3: Modeling of SHG Polarimetry**

The SHG polarimetry from *a*_1_/*a*_2_ nanotwin domain structure described in the main text is given below. The model assumes that the laser beam size (~75 $\mu$m) is much larger than the size of the domains (~10 nm). The domains are also assumed to penetrate through the film thickness, so that one can speak of area fractions of domains instead of volume fractions. Four domains are considered: **Domain 1** with polarization $P_{s}\parallel+x$ and area fraction $A_{x}$, **Domain 2** with polarization $P_{s}\parallel-x$ and area fraction $A_{\bar{x}}$ , **Domain 3** with polarization $P_{s}\parallel+y$ and area fraction $A_{y}$, and **Domain 4** with polarization $P_{s}\parallel-y$ and area fraction $A_{\bar{y}}$. At normal incidence, the SHG intensity depends mainly on the relative area fractions of these domains $\delta A_{x}=A_{x}-A_{\bar{x}}$, and $\delta A_{y}=A_{y}-A_{\bar{y}}$. If the incident linear polarization is rotated in the *x*-*y* plane at an angle $\phi$ from the *x*-axis, the SHG intensity in normal incidence is given by:

$I_{x,y}^{2\omega}=K_{1, x,y}\left( \sin^{2} \phi+K_{2,x,y} \cos^{2} \phi\right)^{2}+K_{3,x,y}\sin^{2} 2\phi+K_{4,x,y} \left( \sin^{2} \phi+K_{2,x,y}\cos^{2} \phi\right)\sin2\phi$ (S1)

$\cos^{2} \Gamma=\frac{K_{4,x}^{2}}{4K_{1,x}K_{3,x}}=\frac{K_{4,y}^{2}}{4K_{1,y}K_{3,y}}$, where $\Gamma= \frac{\omega d}{c}\left| n_{x}^{2\omega}-n_{y}^{2\omega} \right|$ (S2)

$\frac{d_{33}}{d_{31}}=K_{2,x}=\frac{1}{K_{2,y}}$ (S3)

$\left( \frac{d_{15}}{d_{31}} \right)^{4}=\frac{K_{3,y}}{K_{1,y}K_{2,y}^{2}}\cdot\frac{K_{3,x}}{K_{1,x}}$ (S4)

$\left( \frac{\delta A_{x}}{\delta A_{y}} \right)^{4}=\frac{K_{3,y}}{K_{1,y}K_{2,y}^{2}}\cdot\frac{K_{1,x}}{K_{3,x}}$ (S5)

where $\Gamma$ is the phase shift of the SHG signal due to a difference in the index $n_{x}^{2\omega}$ along the *x*- versus the index $n_{y}^{2\omega}$ in the *y*- direction, *d* is the thickness of the thin film (100nm in our case), *c* is the speed of light, and *ω* is the fundamental frequency. The *d_ij_* are the nonlinear coefficients and the $K_{1,x}, K_{1,y}, K_{2,x}, K_{2,y}, K_{3,x}, K_{3,y}, K_{4,x}, K_{4,y}$ are eight fitting parameters for the SHG intensity $I_{x}^{2\omega}$ measured with the analyzer along the *x-* direction or $I_{y}^{2\omega}$ for the analyzer along the *y*- axis. The relations among those fitting parameters described by equations S2 and S3 provide constraints on the fitting parameters. Equations S3 and S4 yield *intrinsic* material constants independent of the exact domain fractions sampled by the laser beam. Equation S2 yields average refractive index birefringence between the *x*- and the *y*-directions. Equation S5 yields information about domain populations, namely the ratio of the net ferroelectric poling in the *x*-direction to that in the *y*-direction. These are plotted in **Figure S5** for the sample influenced by a THz pulse incident polarized parallel to the *x*-direction at time *t*=0s. The polarimetry plots are fitted at various time delays in panel (a). The ratio $\frac{\delta A_{x}}{\delta A_{y}}$ in panel (b) indicates that there is poling in the *x*-direction immediately after the arrival of the THz pulse, followed by a relaxation back to its original domain fraction population at ~160ps. This transient poling under the THz pulse is clearly seen in **Figure S5** panel (b) where the change in the SHG intensity is plotted at the peak of THz excitation, which can be fit to a *single domain* tetragonal microstructure with its ferroelectric polarization in the *x*-direction. The optical birefringence between the *x-* and the *y-* directions in panel (b) also changes by 20% during the presence of the THz pulse and recovers back to its original value over longer period of time. The ratio of nonlinear coefficients, $\frac{d_{33}}{d_{31}}$ increases by over 90% during the THz pulse excitation followed by a recovery to its original value. The ratio of nonlinear coefficients, $\frac{d_{15}}{d_{31}}$ decreases by ~18% during the THz pulse excitation followed by a recovery to its original value.


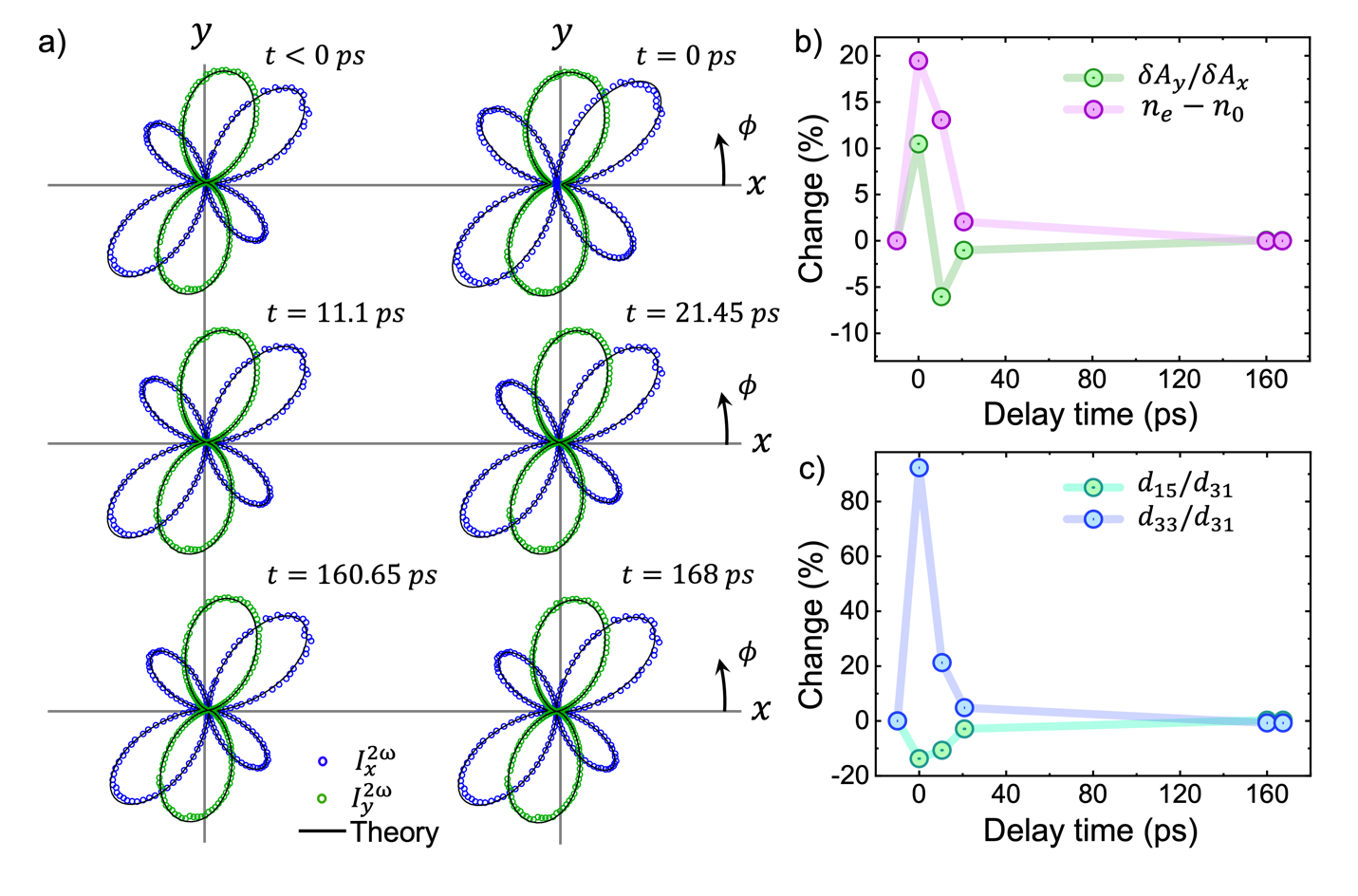


**Figure S5 Time evolution of SHG polarimetry and the corresponding changes in domain area fraction, refractive indices, and SHG coefficient ratios** Time evolution of the SHG polarimetry (a) and the corresponding quantities extracted from SHG modeling in (b) and (c). The polarimetry at t < 0 ps in (a) is from static SHG, while the following ones are from the dynamical SHG which are induced by the THz pump at different time delay. (b) and (c) show the field-induced changes compared to the fitting values of the static SHG.

**
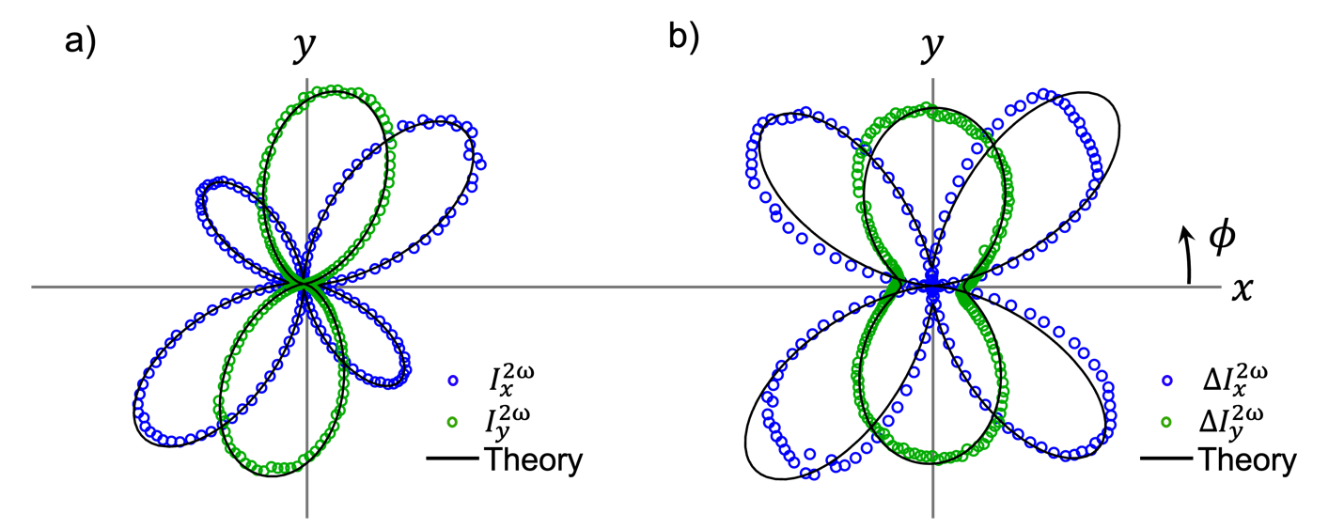
**

**Figure S6 THz field-induced change in SHG polarimetry compared with the static polarimetry (**a) The experimental static SHG polarimetry (circles) and model fit (lines) normalized to unity for analyzer polarization directions along x and y. (b) Experimental data (circles) and 4mm single domain fits (lines) of the THz field-induced change in SHG polarimetry at t = 0. The data and fits are normalized relative to a) and the x and y amplitude scale corresponds to 0.3 relative to unity in a). This result indicates a uniform THz induced dynamic poling effect overlaid on the static a_1_/a_2_ nano-twin domain structure.


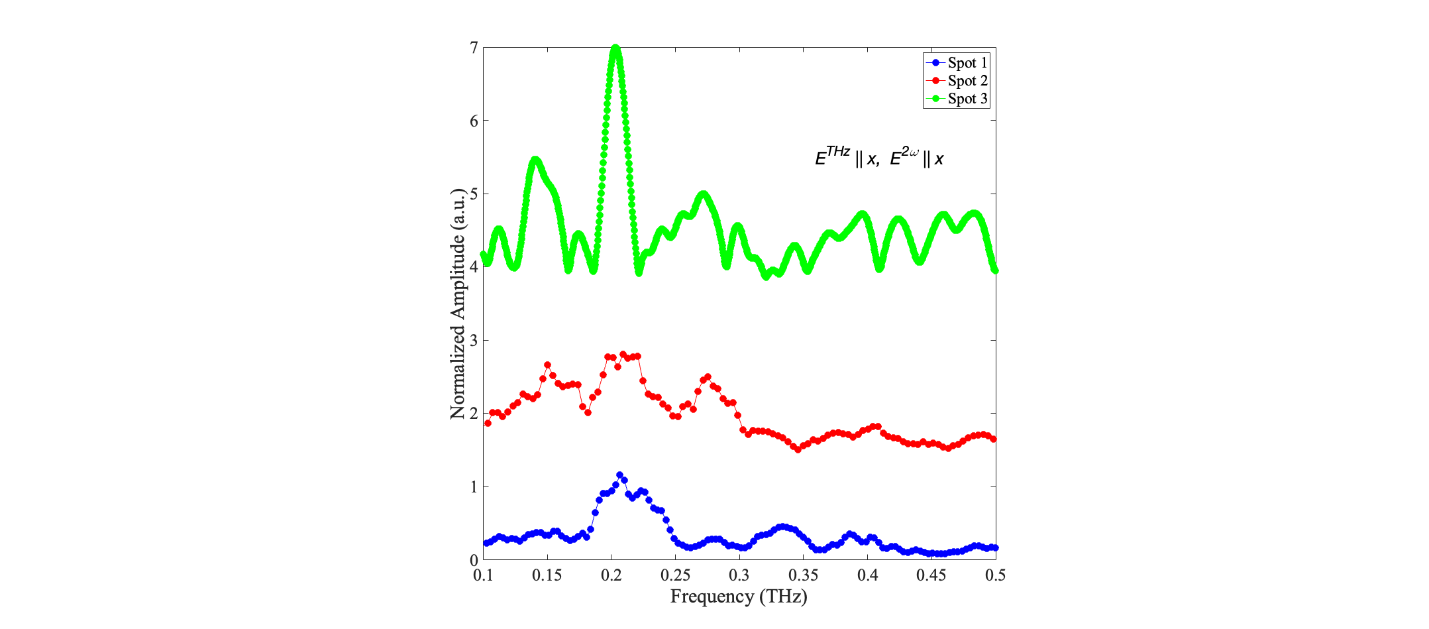


**Figure S7** The FFT of the time-resolved THz-SHG probe measurements when the terahertz field is parallel to the x direction, while the fundamental and second harmonic probe polarizations are both along the x direction in the PTO-STO superlattice film on TSO substrate. The three different curves are from three different film spots with sample moving laterally and a spot spacing of about 200 μm apart. The dominant collective modes are probed consistently in different spots on the sample. However, the experimental amplitude of the modes varies from spot to spot, which is assigned to microstructural variations across the sample surface that modifies the sensitivity to different a_1_/a_2_ nano-twin variants. While a strictly equal population of a_1_/a_2_ nano-twin variants leads to cancellation of net time-resolved SHG probe signal, small imbalance of relative populations of a_1_/a_2_ nano-twin variants allows to detect the collective modes.


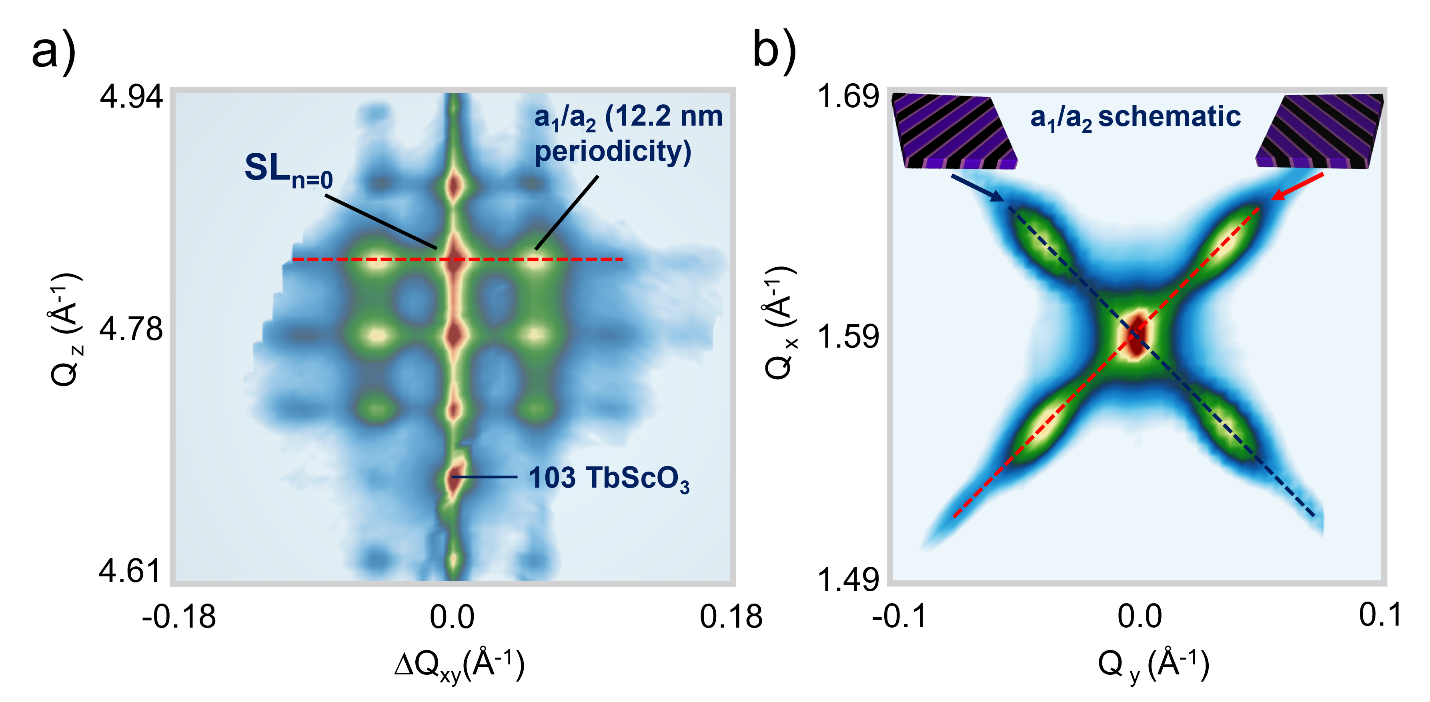


**Figure S8 Reciprocal space (RSM) imaging of a_1_/a_2_ microstructure** a) Diffuse scattering satellites peaks around the 013 TbScO_3_ Bragg reflection, marked along the red dotted line, reveal the periodic nanotwin phase of a_1_/a_2_ along the [110] pseudocubic direction, whereas x and y are along the pseudocubic [100] and [010], respectively. b) Distinct nanotwin variants with different orientation of domain walls are captured in the in-plane RSM cut along the dotted red line from a). The dotted blue and red lines capture satellite peaks from different a_1_/a_2_ nano-twin variants as indicated in the inset. The domain walls of the a_1_/a_2_ nano-twin variants are perpendicular to the dotted lines that intersect the satellite peaks.

**
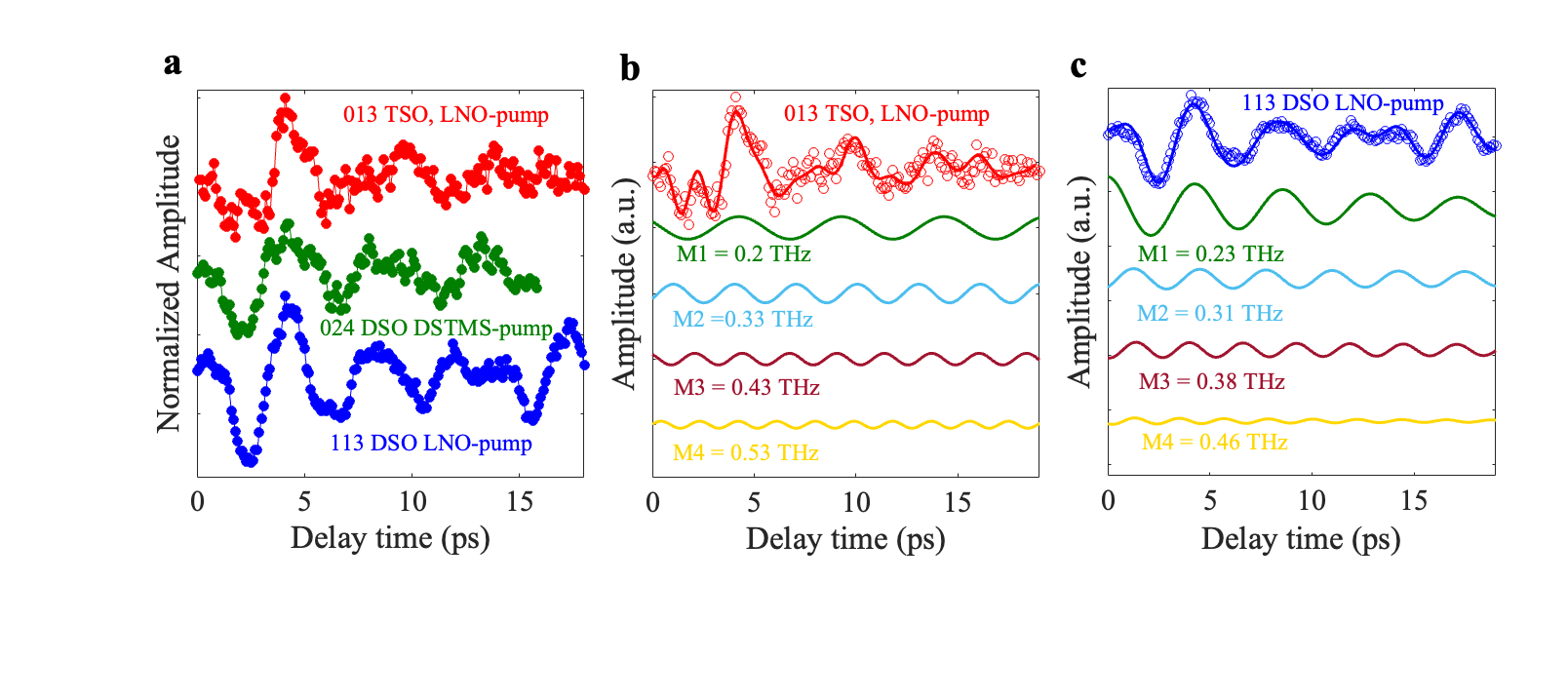
**

**Figure S9 X-ray free electron laser measurements of a1/a2 dynamics on different substrates and using different pulsed THz excitation sources** (a) THz-pump XFEL-probe experiments on a_1_/a_2_ superlattices with various THz sources, diffraction conditions, and different substrates. TSO: TbScO_3_. DSO: DyScO_3_ substrate. LNO: LiNbO_3_ crystal. DSTMS: organic crystal. (b) and (c) Decomposition of collective excitations into four different modes (M_1_, M_2_, M_3_, and M_4_ modes discussed in the main text) for a_1/_a_2_ phase on TSO and DSO substrate, respectively.


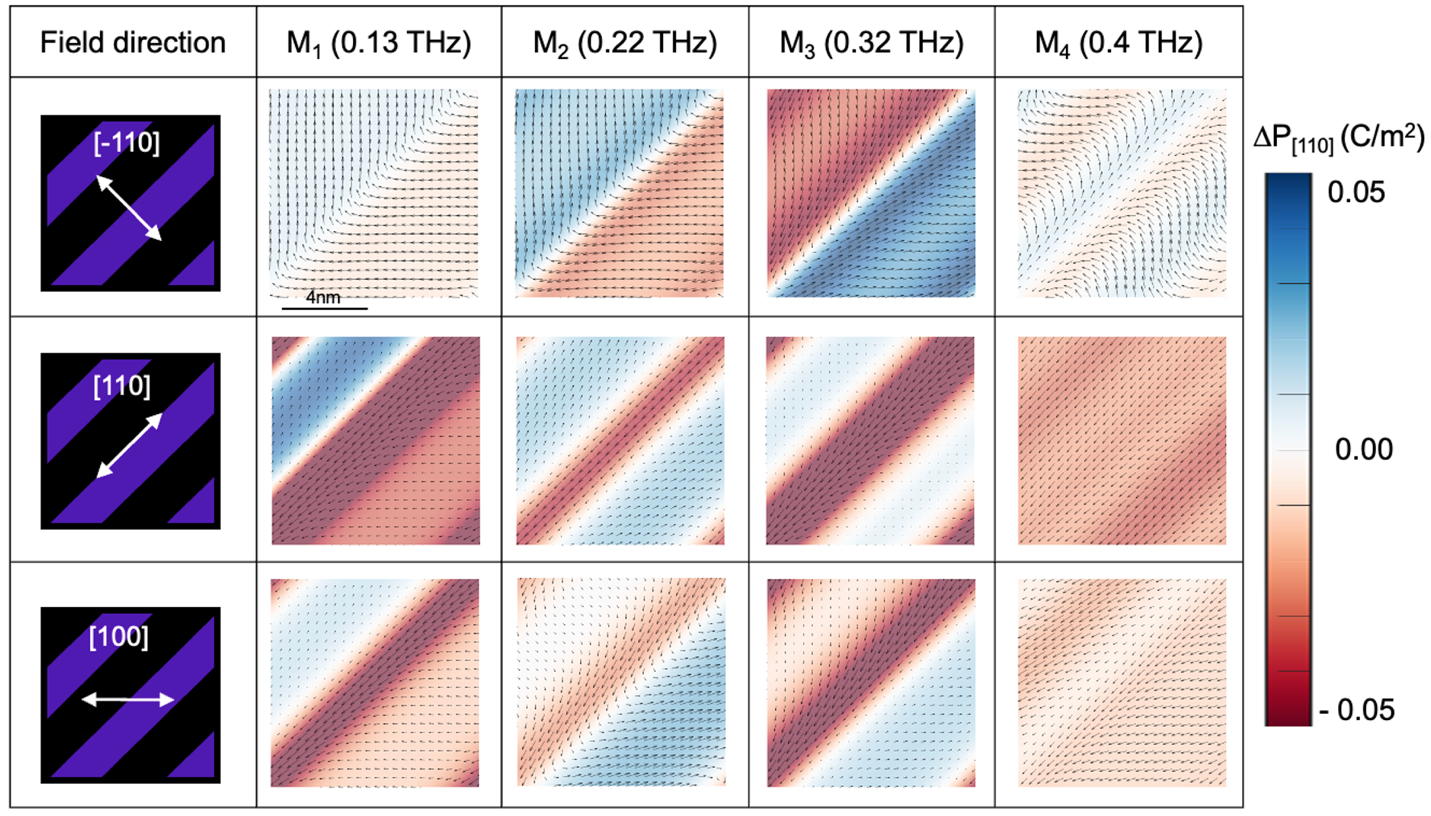


**Figure S10 DPFM simulations of collective modes under specific single frequency excitation** Four representative resonantly excited collective modes predicted by dynamical phase-field modeling (DPFM) a_1_/a_2_ nanotwins arising from THz electric field excitation (right columns) in the directions shown in the column at the left-most column relative to the a_1_/a_2_ nanotwins structure.

**Note 4: Material Coefficients from Phase-Field Simulations**

| Coefficient | PbTiO_3_ ^[48]^ | SrTiO_3_ ^[49]^ |
| --- | --- | --- |
| $a_{11}$ | $3.8\times{10}^{5}(T-T_{c})\left( \frac{J}{m^{3}} \frac{m^{4}}{C^{2}} \right)$  $T_{c}=752 K$ | $7.50\times{10}^{5}T_{s}\left( \coth\left( \frac{T_{s}}{T} \right)-\coth\left( \frac{T_{s}}{T_{c}} \right) \right)\left( \frac{J}{m^{3}} \frac{m^{4}}{C^{2}} \right)$  $T_{c}=30 K$  $T_{s}=54K$ |
| $a_{1111}$ | $-7.252\times{10}^{7} \left( \frac{J}{m^{3}} \frac{m^{8}}{C^{4}} \right)$ | $1.70\times{10}^{9} \left( \frac{J}{m^{3}} \frac{m^{8}}{C^{4}} \right)$ |
| $a_{1122}$ | $7.50\times{10}^{8}\left( \frac{J}{m^{3}} \frac{m^{8}}{C^{4}} \right)$ | $3.92\times{10}^{9}\left( \frac{J}{m^{3}} \frac{m^{8}}{C^{4}} \right)$ |
| $a_{111111}$ | $2.606\times{10}^{8}\left( \frac{J}{m^{3}} \frac{m^{12}}{C^{6}} \right)$ | $0\left( \frac{J}{m^{3}} \frac{m^{12}}{C^{6}} \right)$ |
| $a_{111122}$ | $6.10\times{10}^{8}\left( \frac{J}{m^{3}} \frac{m^{12}}{C^{6}} \right)$ | $0\left( \frac{J}{m^{3}} \frac{m^{12}}{C^{6}} \right)$ |
| $a_{112233}$ | $-3.70\times{10}^{9}\left( \frac{J}{m^{3}} \frac{m^{12}}{C^{6}} \right)$ | $0\left( \frac{J}{m^{3}} \frac{m^{12}}{C^{6}} \right)$ |
| $c_{11}$ | $180\times{10}^{9} (Pa)$ | $180\times{10}^{9} (Pa)$ |
| $c_{12}$ | $80\times{10}^{9}(Pa)$ | $80\times{10}^{9}(Pa)$ |
| $c_{44}$ | $110\times{10}^{9}(Pa)$ | $110\times{10}^{9}(Pa)$ |
| $Q_{11}$ | $0.089 \left( m^{4}/C^{2} \right)$ | $0.0457 \left( m^{4}/C^{2} \right)$ |
| $Q_{12}$ | $-0.026 \left( m^{4}/C^{2} \right)$ | $-0.0135 \left( m^{4}/C^{2} \right)$ |
| $Q_{44}$ | $0.034 \left( m^{4}/C^{2} \right)$ | $0.0096 \left( m^{4}/C^{2} \right)$ |
| $G_{11}$ | $1.04\times{10}^{-10} \left( J m^{3}/C^{2} \right)$ | $1.04\times{10}^{-10} \left( J m^{3}/C^{2} \right)$ |
| $G_{12}$ | $0 \left( J m^{3}/C^{2} \right)$ | $0 \left( J m^{3}/C^{2} \right)$ |
| $G_{44}$ | $0.52\times{10}^{-10} \left( J m^{3}/C^{2} \right)$ | $0.52\times{10}^{-10} \left( J m^{3}/C^{2} \right)$ |
| $\rho$ | $7.5\times{10}^{3}\left( Kg/m^{3} \right)$ | $7.5\times{10}^{3}\left( Kg/m^{3} \right)$ |
| $\beta$ | $6.0\times{10}^{-12}\left( s \right)$ | $6.0\times{10}^{-12}\left( s \right)$ |
| $\kappa^{b}$ | $20 (unitless)$ | $20 (unitless)$ |
| $\mu$ | $9.71\times{10}^{-17}\left( \frac{Kg}{m}\frac{m^{4}}{C^{2}} \right)$ | $9.71\times{10}^{-17}\left( \frac{Kg}{m}\frac{m^{4}}{C^{2}} \right)$ |
| $\gamma$ | $5.88\times{10}^{-6}\left( \frac{Kg}{ms}\frac{m^{4}}{C^{2}} \right)$ | $5.88\times{10}^{-6}\left( \frac{Kg}{ms}\frac{m^{4}}{C^{2}} \right)$ |

**Table S1 |** Phase fields simulation parameters. The polarization mass coefficient ($\mu$) is adjusted to provide a better quantitative agreement to the experimentally observed collective mode frequencies.


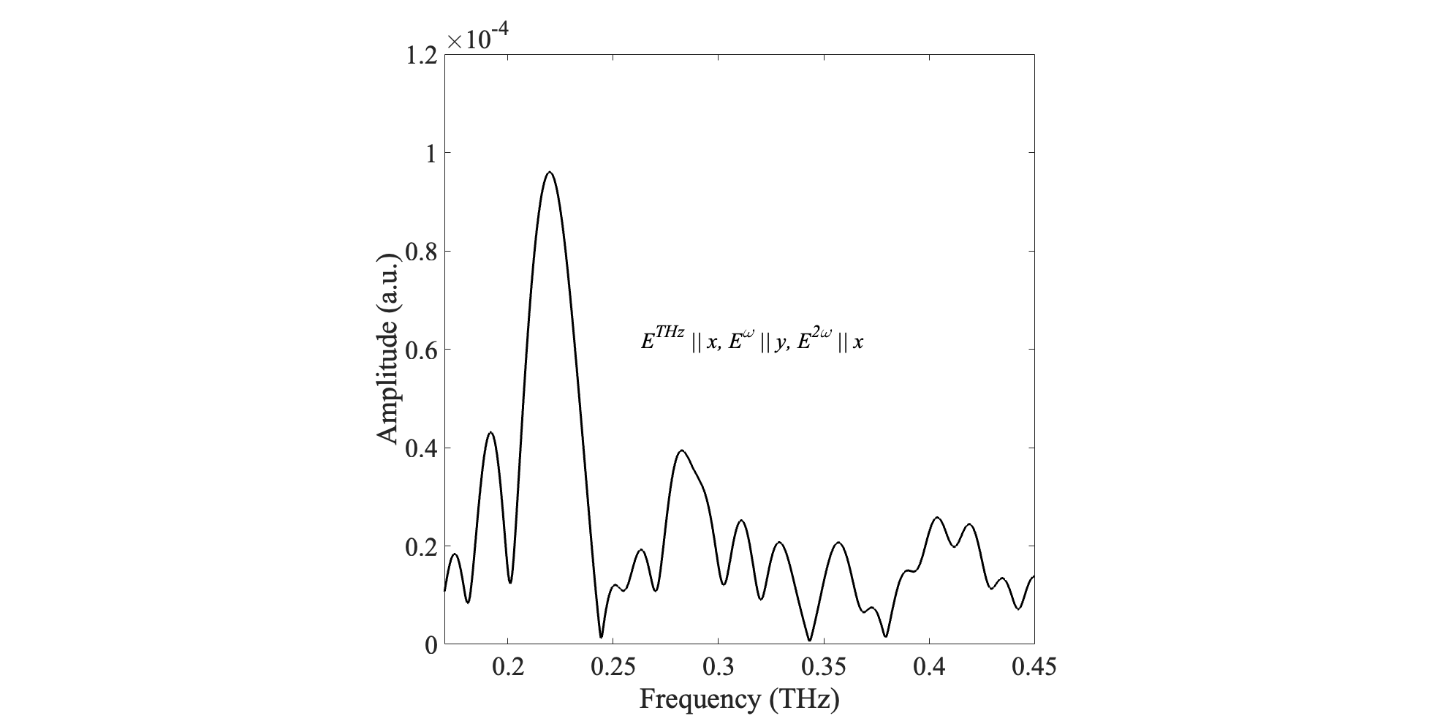


**Figure S11:** **Additional collective modes** captured in the experimental FFT with additional spectral content between the frequencies of M_3_ and M_4_ modes by using different polarization configurations as compared to Fig. 2d) for the a_1_/a_2_ superlattice films on TbScO_3_ substrate. The FFT of the time-resolved THz-SHG measurement is for the terahertz field is parallel to the x direction, the fundamental probe polarization along the y direction and the second harmonic probe polarization along the x direction.

**Note 5: Calculating Domain Wall Velocity Under a Continuous Wave Electrical Field**

We used phase-field simulations to explore how the domain wall motion behavior scales with the amplitude of the applied continuous wave electrical field with the form $E_{i}=E_{0}\sin\left( 2\pi f t \right)\left( \begin{matrix} 1 \\ -1 \\ 0 \end{matrix} \right)$ where $E_{0}$ denotes the amplitude of the electrical field, and $f$ denotes the frequency. For these simulations the frequency was chosen to match the resonance frequency of the *a_1_/a_2_* breathing mode ($f=0.13 THz)$. To track the domain wall position, we assumed that the center of the *a_1_/a_2_* domain wall is the position which minimizes $|P_{1}+P_{2}|$.


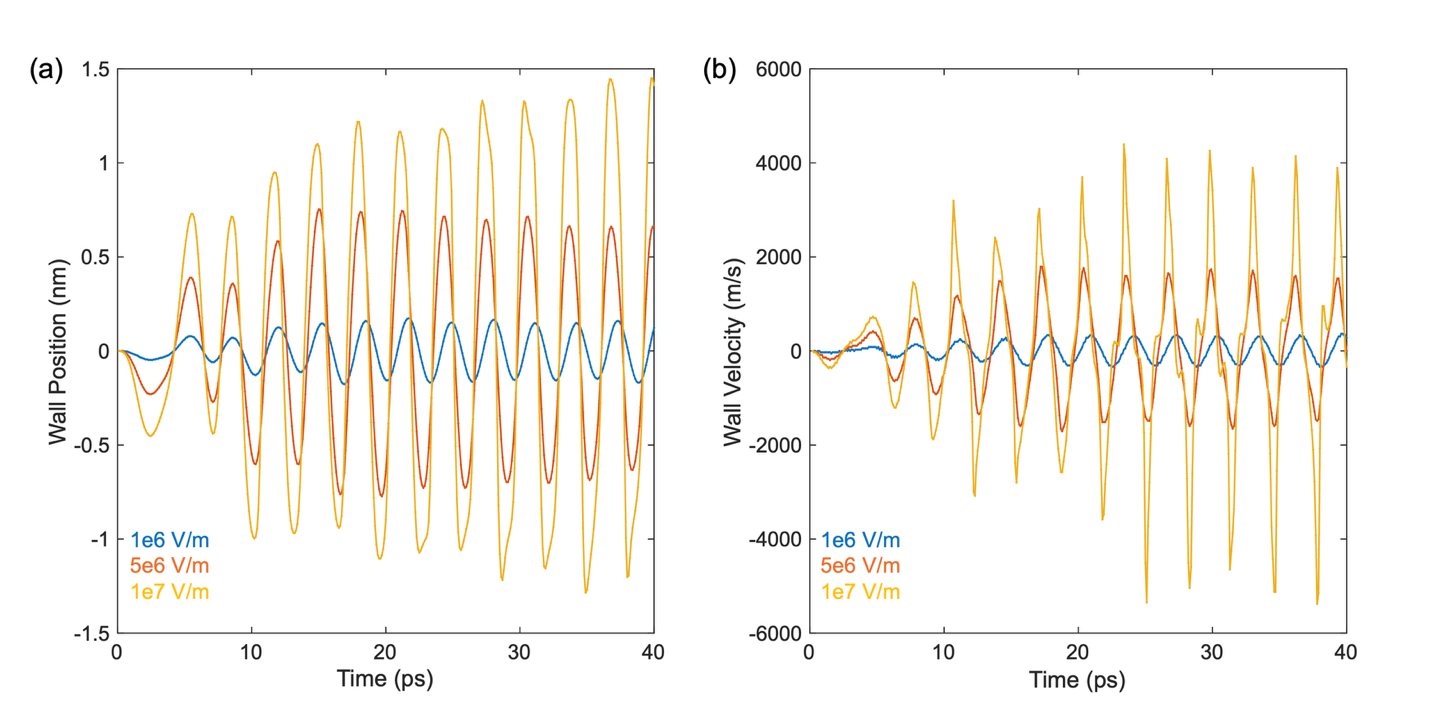


**Figure S12: Resonantly Excited Domain Wall Dynamics of a₁/a₂ Domains**. (a) Time-dependent evolution of the domain wall position under different applied electric fields. (b) Corresponding time-dependent domain wall velocity under different applied electric fields.

**Figure S12,** shows the time dependent response of the resonantly excited *a_1_/a_2_* breathing mode. Starting from rest, the domain walls require approximately two oscillation periods for the amplitude of the domain wall motion to saturate. After the motion saturates, the domain wall motion behavior remains constant with only minor oscillations in the peak domain wall amplitude. To further quantify the dynamics, **Table S2** summarizes the dependence of the domain wall motion of the applied electric field, showing both the wall amplitude and the corresponding domain wall velocities. To calculate the averages, the wall position amplitude and peak wall velocity are sampled between 20 and 40 ps. The uncertainties represent 1 standard deviation.

**Table S2 |** Electric field dependence of domain wall position and peak domain wall velocity

| Electric Field $(E_{0})$ | Wall Position Amplitude | Peak Wall Velocity |
| --- | --- | --- |
| 1e^6^ V/m | 0.15 nm ± 0.01 | 316 ± 20 m/s |
| 5e^6^ V/m | 0.70 nm ± 0.04 | 1360 ± 285 m/s |
| 1e^7^ V/m | 1.24 nm± 0.12 | 4422 ± 626 m/s |

**References:**

[20] A.R. Damodaran, J.D. Clarkson, Z. Hong, et al., *Nature Materials* 16 (2017), 1003–1009**.**

[48] M. J. Haun, E. Furman, S. J. Jang, H. A. McKinstry, L. E. Cross, *Journal of Applied Physics* 62 (1987), 3331.

[49] G. Sheng, Y. L. Li, J. X. Zhang, S. Choudhury, Q. X. Jia, V. Gopalan, D. G. Schlom, Z. K. Liu, L. Q. Chen, *Applied Physics Letters* 96 (2010), 232902.
